# Investigation on a mobile fire extinguishing approach using liquid carbon dioxide as inert medium for underground mine

**DOI:** 10.1371/journal.pone.0299940

**Published:** 2024-04-15

**Authors:** Liang Ge, Zujing Zhang, Yinjun Wang, Shitao Zhang, Yujin Chen

**Affiliations:** 1 State Key Laboratory of Mechanical Transmission for Advanced Equipment, Chongqing University, Chongqing, China; 2 State Key Laboratory of Coal Mine Disaster Prevention and Control, China Coal Technology and Engineering Group Corp Chongqing Research Institute, Chongqing, China; 3 College of Civil Engineering, Guizhou University, Guiyang, China; 4 Chongqing Key Laboratory of Manufacturing Equipment Mechanism, Chongqing Technology and Business University, Chongqing, China; Tianjin University, CHINA

## Abstract

Injecting carbon dioxide is the most effective means of preventing and extinguishing fires in sealing hazardous areas, but the traditional method slowly and remotely injects carbon dioxide gas into the well after gasification on the ground, which is dependent on the complete mine pipe network without cooling effect. To inject liquid directly from the tank with vacuum interlayer and heat insulating powder for rapid inerting and cooling, a new approach using track mobile platform to go deep into the underground mine disaster area is proposed, so the liquid can be delivered to the nozzle at the end of DN40 large diameter pipe, and the continuous gasification jet can be realized. The experimental results show that: (1) The liquid volume in a tank of vacuum degree within 2.0 Pa and 200 mm interlayer reduced no more than 15.5% after 48 days; (2) Taking the pressure in the tank as the power source, because of environmental differences inside and outside the pipe after 100 m pressure holding delivery, the physical form of liquid and gas could be converted instantly; (3) The continuous discharge time without ice blocking for a tank full of 2 m^3^ liquid was about 10.5 min under 25 L dual mode nitrogen pressurization, which is 1/12 of injection time after ground gasification; (4) Based on the temperature decrease trend measured at different positions, the cooling characteristics on liquid gasification jet path are quantified, and the calculation formula of temperature changing with time on the center line of liquid gasification jet is obtained. Through this new approach, the integration of vacuum insulated storage, safe mobile transportation, and continuous and rapid release with large flow can be achieved for the liquid carbon dioxide.

## Introduction

Mechanization level and production efficiency of coal mine was greatly improved [1] with vigorous promotion and application of top coal caving technology and mining method, but because of large creeping height, more coal left in the goaf, serious air leakage [2, 3], so the spontaneous combustion of coal mine was frequent to become one of common disasters [4] and widely threat the industry of coal mining [5, 6]. Mine fires due to spontaneous combustion of coal is a major problem in the leading coal producing countries like China, USA, India, Australia, Germany, etc [7]. In China, mines with spontaneous combustion account for 51% of all major coal mine accidents, and more than 60% of mine fires result from spontaneous combustion in goaf [8, 9]. In addition, a high temperature fire point under coal mine is a major hazard source that can easily lead to the fire or the explosion [10, 11] mainly caused by the methane [12], which is accompanied by the accumulation of toxic gases. Therefore, research on technology and equipment of fire prevention and extinguishing are very concerned greatly by domestic and foreign scholars [13], isolation of coal or other burning materials from oxygen and dropping the temperature of coal or other burning materials are used as the main methods [14, 15], such as grouting [16, 17], water mist [18], colloid [19], inert gases [20], cellular grout [5] and cryogenic slurry [21], have been developed and applied throughout the world. However, grouting using a considerable amount of slurry, water mist, colloid consisted of sodium silicate and ammonium salt cannot extinguish the large scale fires due to poor coverage [22], limited jet range [16], high cost and a toxic gas ammonia given off [10] corresponding. Injecting the inert gas into the fire zone is the most effective mean used to control the disaster, there are various types of injection equipment for mining inert media, and the comparison is shown in Table 1.

**Table 1:** Comparison of injection equipment for mining inert media.

Equipment name	Production mechanism	Gas production (m^3^/h)	Equipment unit price (RMB)	Gas production cost (RMB/m^3^)	Technical performance
Fuel inert gas fire extinguishing device	Generate inert gas by burning kerosene.	1 000	1.20 million	3.00	Gas contains a small amount of oxygen and carbon monoxide, resulting in poor safety, which has been eliminated.
Nitrogen generator	Gas production by air separation method.	1 000	2.00 million	2.00	High outlet temperature and high maintenance frequency; The maximum oxygen content in membrane separation gas production is 5%.
Carbon-dioxide generator	Gas production from the reaction of concentrated sulfuric acid and sodium bicarbonate.	1 000	0.85 million	8.00	Chemical reaction production has low safety, low gas production, and a purity of 98%.
Liquid carbon dioxide gasification and injection system	Chemical reaction production has Low safety, low gas production, and a purity of 98%.	1 170	0.80 million	1.80	Direct injection of liquid can suffocate, cool down, dilute gas, suppress explosion, and Prevent reignition in fire areas; Chemical plants produce liquids with a purity of nearly 100%, providing strong resource guarantee.
Liquid carbon dioxide direct injection device		5 850	1.00 million		

The first case of liquid nitrogen adopted in fire extinguishing was in 1949, in the Doubrave coal mine of the Czech Republic. As an inert medium, the carbon dioxide is mainly produced through industrial waste liquid and exhaust gas. Based on the secondary recycling and utilization of industrial emissions, it is transformed into a disaster prevention and control measure that effectively reduces property losses in high-risk workplaces and ensures the safety of workers, it is also an important embodiment of the concept of green manufacturing and environmental protection. Using carbon dioxide without residue and corrosion as well as conductivity in coal mines is obvious for inerting effect, in addition of preventing combustion and explosion, the equipment purchase and maintenance costs are low [23]. Therefore, this technology and equipment of fire prevention and extinguishing or toxic gases diluting has good economic and social, as well as environmental benefits, which is widely used and gradually replaced other types of inert gas.

**Fig 1:**
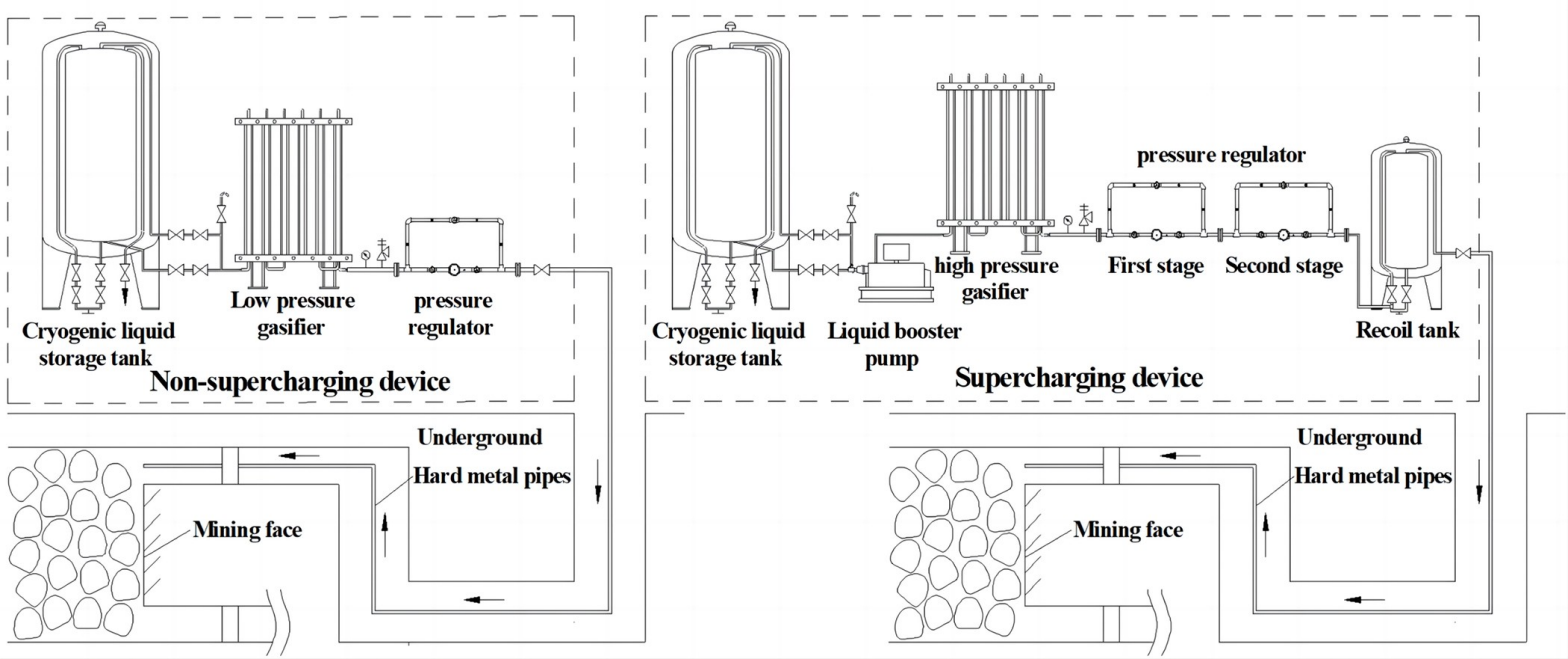
Structure and composition of gasified and regulated device.

At present, the liquid carbon dioxide gasified and regulated in the mine ground is transported remotely to the underground mine disaster area by pre-installed hard metal pipes with the diameter of 100 mm~150 mm. According to the actual conditions of mine construction, the pipe laying and the actual needs of terminal pressure and flow rate, it can be divided into two types, namely non-supercharging and supercharging types, as shown in Fig 1. This method can achieve the emergency treatment to fire accidents and has been used by many countries, such as Germany, the former Soviet Union and other countries. In June 2011, the same method was carried out in the Yuwu coal mine of Lu’an Coal Industry (Group) Co., Ltd. through ground drilling, which was the first in China and achieved good results. However, the above successful application cases were dependent on the construction situation of existing pipelines using for pressure ventilation, grouting, drainage and injecting inert gas, but about 90% of coal was produced in underground mining in China [24], and most of production mines did not have the complete pipeline systems, especially in the case of catastrophic damage. In addition, using this method to make the gas into the fire zone, the role of cooling would be weakened, and the accident of long pipe network would be easily caused by the dry ice generated near the release orifice [25], while the ground control and the underground disaster area monitoring would be too difficult to coordinate in time. Therefore, carbon dioxide application for serious mine fires or mine toxic gases seems to be a good idea but still needs to be improved to go deep into the underground mine disaster area, uses its own pipeline, and directly inject liquid into the area to be inerted.

In recent years, a small number of institutions carried out related technology research on underground direct injection of liquid carbon dioxide based on the actual situation of coal mining in China and disaster characteristics. Similar equipment are mainly to simply emulate the overall structure and function of ground inflatable station, but they are not suitable for mine disaster environment because of some technical problems, such as: (1) The short liquid storage time; (2) The self-boost control technology with poor reliability and only a single operating mode; (3) The low flow liquid delivery pipe without low temperature resistance, corrosion resistance and aging resistance; (4) The completeness of valve functions, automation operation and other safety technical measures need to be improved; (5) The test situation of performance influencing factors and the application effects of research results have not been reported.

In order to solve the shortcomings of existing equipment, the authors studied the principle of liquid direct injection based on breakthroughs in key technologies, and designed the overall light structure using mobile and articulated base applied to the mine track as a mobile platform to achieve the functions of installation and transportation for all components, so the new mobile approach used in underground mine disaster area is built, then the test analysis was carried out. The liquid carbon dioxide from fuel gas mixture in the industrial application [26] shipped by transport vehicles is filled in cryogenic storage tank of vacuum insulation and medium pressure [27], which is used for pressure storage and isolation of external heat. A combination of several sections by the joining of mine locomotive is transported to near the underground mine disaster area. The liquid carbon dioxide is jetted from insulation storage tank to fire zone by using the high intensity pipes after operation of self-pressurized regulating system. Based on rapid gasification and expansion of liquid carbon dioxide jetted to the fire zone, it can cover and inerting fire zone, extinguish the fire source [28] and cool quickly, along with the suppression of gas and coal dust explosion. The new mobile approach can achieve the following effects: (1) The tank filled with perlite powder in the vacuum insulation interlayer allows liquid insulation storage time to exceed 48 days, which is superior to the existing non vacuum insulation structure that only maintains liquid storage for 48 hours; (2) The dual mode nitrogen pressurization control for anti-ice block different from the existing liquid split gasification feedback approach, the new approach has the advantages of large gas flow rate and fast speed as well as materials safety; (3) The new self-compensation system controls the pressure of automatic safety relief at 2.20 MPa, but the existing system compensates for the insufficient capacity of air replenishment and pressurization by increasing the relief pressure to 2.60 MPa, its effect is to sacrifice structural safety to support the entire liquid discharge; (4) The new pipeline network adopts the unique confluence and quick combination of stainless steel pressure pipe, which is convenient for personnel to operate, so it can make up for the shortcomings of existing DN25 rubber pipe in non quick combination switching between pipelines and between tanks; (5) The new construction method of DN40 short-distance continuous liquid delivery realizes the discharge of 2 m^3^ liquid in a single tank within 12.0 minutes, its discharge speed is nearly 1.5 times that of existing DN25 pipeline; (6) The new approach focuses on the safe protection and adaptability to harsh environment, such as collision prevention, dual configuration of key valves, and fixed output pressure of nitrogen gas cylinders.

## Methods

### Technical principles

**Fig 2:**
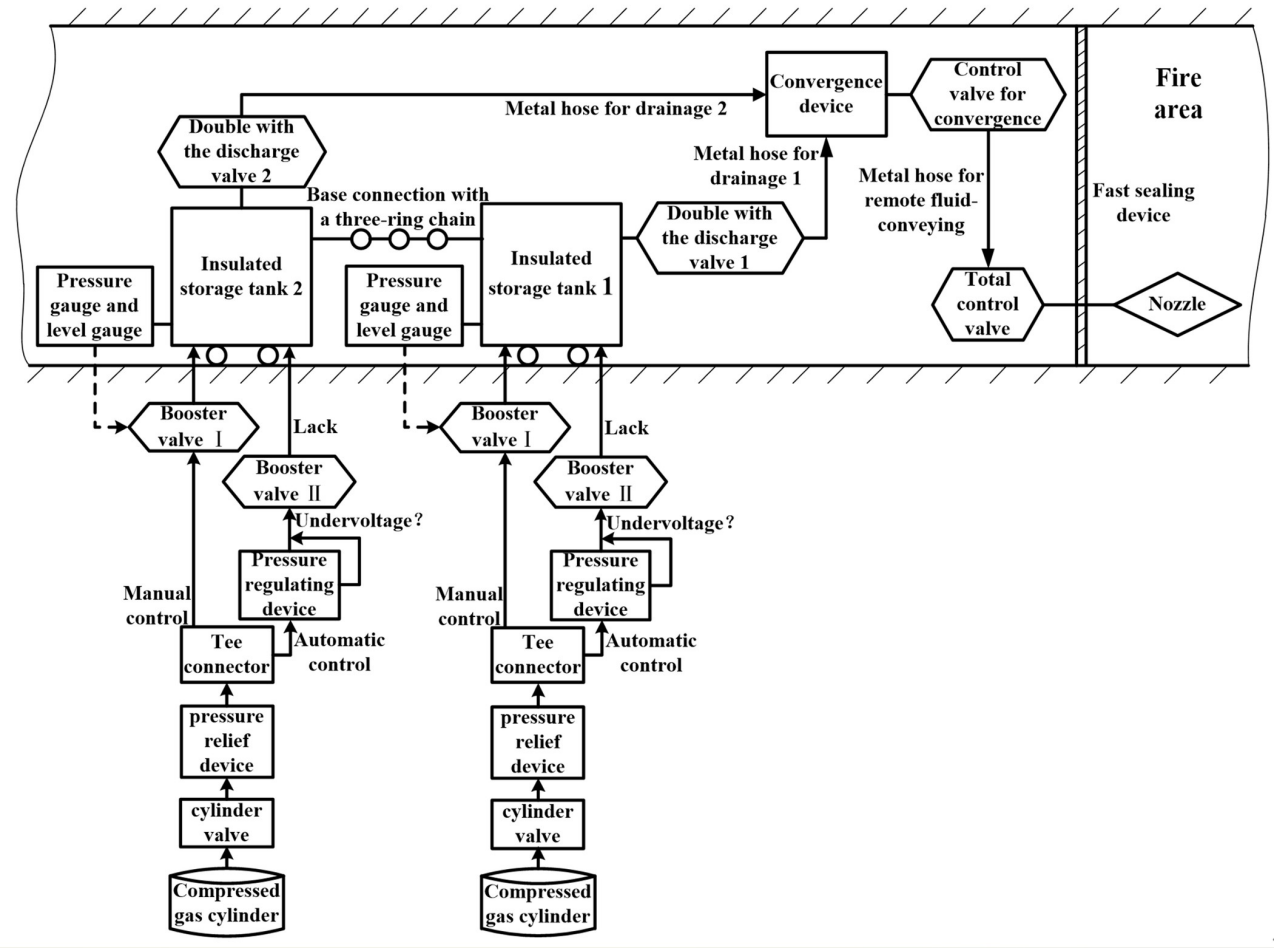
Technical schematic of system application.

Take two tanks in series as an example, the technical principle of new liquid carbon dioxide inerting equipment is shown in Fig 2. When the system works, pressure of liquid carbon dioxide in storage tank is as its driving force to flow to the affected areas by the fluid delivery pipe, and final gasification jet is accomplished by nozzle. When holding liquid discharges of storage tank, in order to maintain the inner cylinder having a pressure of more than 1.20 MPa so as to avoid the formation of liquid into solid ice, the self-pressurization control system should be put into work. High pressure compressed gas in cylinder is depressurized by the pressure relief device to flow into the tee connector, then the gas pressurized delivery is divided into two modes, namely the manual control based on the real-time observation of pressure gauge and the level gauge, and the unattended automatic control based on pressure regulating device. The pressure regulating device adopts principles of controlling outlet pressure, and it can allocate a reasonable amount of supplementary gas automatically to hold constant pressure according to changes of actual pressure in the tank. Connection of tee connector and pressure regulating device as well as gas booster valve I or valve II are realized by metal hose used for air supply and pressure increasing.

### Key technologies

**Horizontal pressure-resistant insulation and automatic pressure relief regulation technology.** The underground mobile storage tank should always store a certain amount of liquid carbon dioxide in a state of combat readiness, which requires the long liquid storage time to avoid the consumption of manpower and costs caused by repeated filling. However, as the liquid inside the tank continues to stand still, which continuously vaporizes and decreases with the pressure growing. It is necessary to solve the problem of how to effectively reduce the liquid loss, in order to minimize the impact of external environmental temperature on the storage temperature inside the tank. When the pressure inside the tank slowly reaches the allowable pressure threshold, it is necessary to solve the problem of quickly preventing over limit, in order to avoid leakage caused by damage to the tank structure.

**Fig 3:**
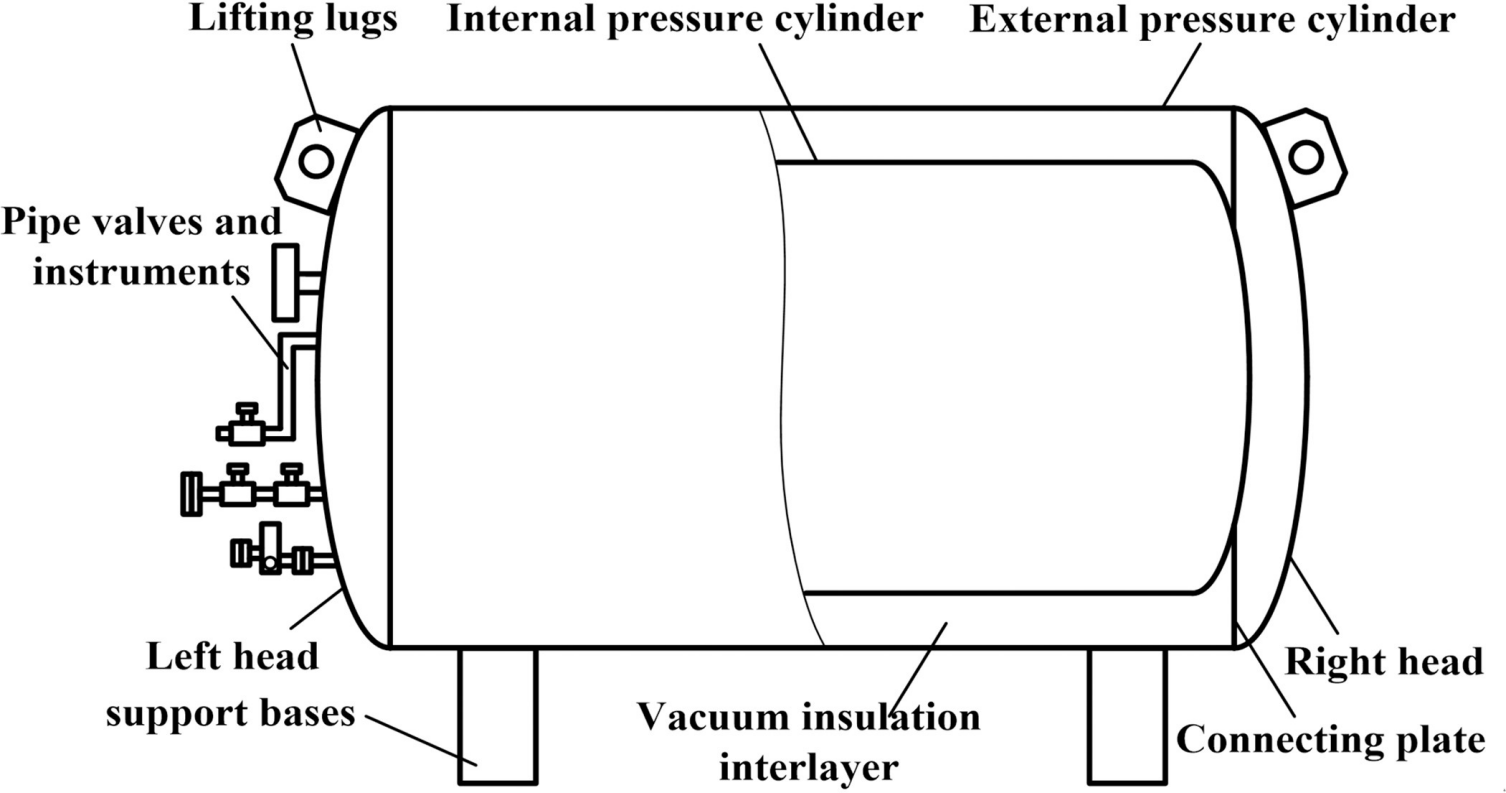
Structure diagram of insulation storage tank.

Fig 3 shows the structure diagram of insulation storage tank, which consists of double layer structure, fitting pipe valves and instruments, and support bases. The double layer structure needs certain structural strength and sealing properties to prevent the toxic environment from the large-scale leakage of carbon dioxide [29], which is made of cylinder and left head as well as right head through welding, where the left head and right head are outwardly protruding arcs. According to the design standard of Class II pressure vessel in China, the Q245R or Q235-B material with nominal thickness of not less than 7 mm is used in the outer structure, and the inner structure is made of 16MnDR material with nominal thickness of not less than 10 mm. Both of them are nested by the connecting plate to form the vacuum insulation interlayer, which is filled with a thermal insulation material called perlite powder, so that the increase in liquid storage time reduces the filling and maintenance costs. After self-enhanced treatment, the use of residual stress is beneficial to improving the pressure capacity and extending the service life. The wall thickness of outer cylinder is increased to improve the impact resistance and the lifting lugs are provided to facilitate the installation of storage tank. The pipe valves and instruments of insulation storage tank include shut-off valves, safety valves, pressure and liquid level indicator, and vacuumize connection device to realize intake and discharge of liquid and gaseous carbon dioxide, full test analysis of liquid, blowdown of residue liquid, monitoring and display of pressure and liquid level in inner storage tank, automatic pressure relief, and gas extraction in interlayer. The safety and reliability of upper inlet liquid valves, lower inlet liquid valves, drain valves, gas pipe valves and relief valves are improved by the way of dual configuration. The multiple independent gas pipe branches are merged to reduce the number of steel pipe between the inner and outer portions of cylinder, further reduce the incoming heat from the outer portion. A saddle-type support structure is connected with the outer cylinder by welding and connected with the mobile and articulated base through bolts. In order to avoid the road surface excitation or the damage caused by the impact of vibration on the cylinder or supporting pipes and valves, damping materials with a certain flexibility or the obvious attenuation characteristics for vibration amplitude is set between the two contact surfaces to enhance the ability to pass in the restricted disaster area environment.

In accordance with the Chinese standards ‘TSG R0004-2009 Fixed pressure vessel safety technical supervision procedures’ and ‘GB 150.1~4–2011 Pressure vessel’, structural models are established in turn, which include the cylinders and the heads as well as the double saddles under the internal and external pressure. Based on the design conditions of steel horizontal pressure vessel, the calculation and check of external pressure cylinder and internal pressure left head as well as double saddle are used as examples.

*(1) External pressure cylinder*. The stress check should be performed in air pressure test, the stress under test pressure *σ*_*T*_ of cylinder is defined as following formula.


$$\sigma_{T}=\frac{P_{T}(D_{i}+\delta_{e})}{2\delta_{e}\emptyset }$$ (1)

Here, *D*_*i*_ denotes the inside diameter, taking 1484.00 mm; *ϕ* denotes the coefficient of welded joint, taking 0.85. The test pressure *P*_*T*_ and the effective thickness *δ*_*e*_ used for thickness evaluation are defined as following formulas.


$$P_{T}=\frac{1.1P_{c}[\sigma ]}{{\left[\sigma \right]}^{t}}$$ (2)

$$\delta_{e}=\delta_{n}‐C_{1}‐C_{2}$$ (3)

Here, *P*_*c*_ denotes the calculated pressure, taking -0.10 MPa; [*σ*] denotes the allowable stress under test temperature, taking 116.00 MPa; [*σ*]^*t*^ denotes the allowable stress under design temperature, taking 114.88 MPa; *δ*_*n*_ denotes the nominal thickness, taking 7.35 mm; *C*_1_ denotes the negative deviation of steel plate, taking 0.30 mm; *C*_2_ denotes the corrosion margin, taking 1.00 mm. The allowable stress levels adopted under pressure test [*σ*]_*T*_ denotes the allowable value of strength check, which can be calculated according to the following formula.


$${\left[\sigma \right]}_{T}\leq 0.8\sigma_{s}$$ (4)

Here, *σ*_*s*_ denotes the yield point at test temperature, taking 235.00 MPa. *σ*_*T*_ is 15.94 MPa calculated by Formulas (1) to (3), [*σ*]_*T*_ does not exceed 188.00 MPa. Hence, the stress under test pressure of cylinder does not exceed its allowable stress levels adopted under pressure test, satisfies the check conditions of *σ*_*T*_≤[*σ*]_*T*_, the parameter selection and design strength are reasonable.

*(2) Internal pressure left head*. Stress under test pressure *σ*_*T*1_ of internal pressure left head is defined as following formula.


$$\sigma_{T1}=\frac{P_{T}(KD_{i}+0.5\delta_{e})}{2\delta_{e}\emptyset }$$ (5)

Here, *D*_*i*_ denotes the inside diameter, taking 1100.00 mm; *ϕ* denotes the coefficient of welded joint, taking 1.00; The coefficient *K* is defined as following formula.


$$K=\frac{1}{6}[2+{\left(\frac{D_{i}}{2h_{i}}\right)}^{2}]$$ (6)

Here, *h*_*i*_ denotes the surface depth, taking 275.00 mm. The test pressure *P*_*T*_ and the effective thickness *δ*_*e*_ as well as the allowable stress levels adopted under pressure test [*σ*]_*T*_ are calculated by the same method with the external pressure cylinder. Therefore, *P*_*T*_ is calculated from the calculated pressure *P*_*c*_ of 2.37 MPa and the allowable stress under test temperature [*σ*] of 181.00 MPa as well as the allowable stress under design temperature [*σ*]^*t*^ of 181.00 MPa by Formula (2); *δ*_*e*_ is calculated from the nominal thickness *δ*_*n*_ of 8.70 mm and the negative deviation of steel plate *C*_1_ of 0 mm as well as the corrosion margin *C*_2_ of 1.00 mm by Formula (3); [*σ*]_*T*_ is calculated from the yield point at test temperature *σ*_*s*_ of 245.00 MPa by Formula (4). The stress under test pressure *σ*_*T*1_ of internal pressure left head calculated by Formula (5) is 187.08 MPa, [*σ*]_*T*_ does not exceed 196.00 MPa. Hence, the stress under test pressure of internal pressure left head does not exceed its allowable stress levels adopted under pressure test, the goal of *σ*_*T*_≤[*σ*]_*T*_ is reached.

*(3) Double saddle*. In order to calculate and check the circumferential stress of cylinder at the saddle, it is necessary to calculate the bearing reaction force *F* by the following formulas.


$$\left\{ \begin{array}{c} F=\max\left(F_{1},F_{2}\right)\\ F_{1}=\frac{1}{2}(m_{1}+{2m}_{2}+m_{3}+m_{4a}+m_{5})g\\ F_{2}=\frac{1}{2}(m_{1}+{\mathrm{2m}}_{2}+m_{3}+m_{\mathrm{4b}}+m_{5})g \end{array}\right.$$ (7)

Here, *F*_1_ denotes the bearing reaction force during operation; *F*_2_ denotes the bearing reaction force during pressure test; *m*_2_ denotes the head mass (surface part), taking 94.20 kg; *m*_3_ denotes the accessory mass, taking 0 kg; *m*_5_ denotes the heat resistant layer quality, taking 0 kg; *g* denotes the gravitational acceleration. The cylinder mass (between two tangent lines) *m*_1_, the total mass at work *m*_4a_, and the total mass during the pressure test *m*_4b_ are calculated by the following formulas.


$$m_{1}=\pi (D_{i}+\delta_{n})L_{c}\delta_{n}\gamma_{s}$$ (8)

$$m_{4a}=V\gamma_{0}\phi_{0}$$ (9)

$$m_{4b}=V\gamma_{T}$$ (10)

Here, *L*_c_ denotes the distance between the tangent of two heads, taking 2400 mm; *γ*_s_ denotes the density of cylinder, taking 7.85×10^−6^ kg/mm^3^; *γ*_0_ denotes the material density during work, taking 0.6×10^−9^ kg/mm^3^; *Φ*_0_ denotes the material filling coefficient, taking 0.95; *γ*_*T*_ denotes the medium density of hydraulic test, taking 1×10^−6^ kg/mm^3^; *V* denotes the volume of container (between the two tangent lines), taking 4.15×10^9^ mm^3^. Since the cylinder has no reinforcing ring and is reinforced by the backing plate, the circumferential stress *σ*_1_ at the lowest point of cross section, the circumferential stress *σ*_2_ at the saddle corner, and the circumferential stress *σ*_3_ in the cylinder at the edge of saddle pad are calculated as following formulas.


$$\left\{ \begin{array}{c} \sigma_{1}=‐\frac{kK_{1}F}{(\delta_{e}+\delta_{\mathrm{re}})b_{1}}\\ \sigma_{2}=‐\frac{F}{4(\delta_{e}+\delta_{\mathrm{re}})b_{1}}‐\frac{\mathrm{12}K_{2}FR_{a}}{L_{C}(\delta_{e}^{2}+\delta_{\mathrm{re}}^{2})}\\ \sigma_{3}=‐\frac{F}{4\delta_{e}b_{1}}‐\frac{\mathrm{12}K_{3}FR_{a}}{L_{C}\delta_{e}^{2}}\\ b_{1}=b+\mathrm{1.56}\sqrt{R_{a}\delta_{n}} \end{array}\right.$$ (11)

Here, *δ*_re_ denotes the effective thickness of saddle pad, taking 8 mm; *k*, *K*_1_, *K*_2_ and *K*_3_ denote the correlation coefficients, taking 0.109, 0.760, 0.013 and 0.011 respectively; *b*_1_ denotes the effective width of cylinder; *b* denotes the axial width of saddle, taking 200 mm; *R*_*a*_ denotes the average radius of cylinder, taking 749.35 mm.

The inside diameter *D*_*i*_ and the allowable stress under design temperature [*σ*]^*t*^ as well as the effective thickness *δ*_*e*_ refer to the calculation and check of external pressure cylinder. Firstly, *m*_1_, *m*_4a_ and *m*_4b_ can be calculated respectively as 648.451 kg, 2.366 kg and 4150 kg by Formulas (8) to (10); Secondly, *F* is 24435.570 N calculated by substituting into formula group (7); Furthermore, *σ*_1_, *σ*_2_ and *σ*_3_ are -0.46 MPa, -13.21 MPa and -30.71 MPa calculated by substituting into formula group (11). Therefore, |*σ*_1_|<[*σ*]^*t*^, |*σ*_2_|<1.25[*σ*]^*t*^, |*σ*_3_|<1.25[*σ*]^*t*^, the design strength of double saddle satisfies check requirements for circumferential stress of cylinder at the saddle.

#### Self-compensation pressurization technology

When the pressure inside the insulated storage tank and in the discharge pipeline is lower than the critical point of ice blockage, liquid carbon dioxide will solidify into dry ice and cannot continue to be transported and gasified. It is necessary to solve the problem of whether the entire discharge process from full liquid to discharge can be uninterrupted. Timely compensation should be made based on the degree of insufficient pressure, and the delivery pressure drop on multiple combined pipelines should be effectively reduced.

**Fig 4:**
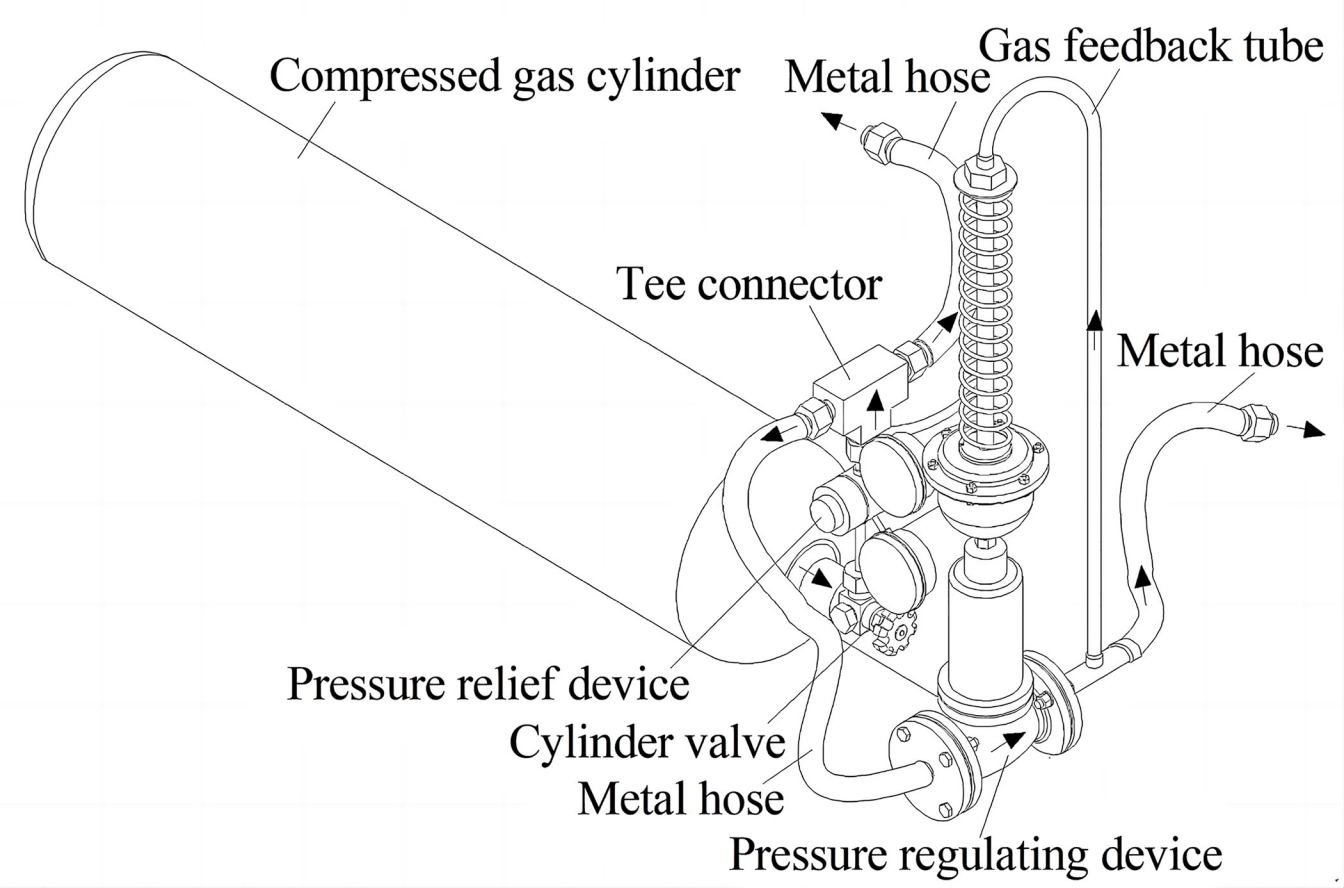
Structure diagram of self-pressurization control system.

The self-pressurization control system for anti-ice is composed of compressed gas cylinder with the cylinder valve, pressure relief device, tee connector and pressure regulating device, the combination of stainless steel metal hose forms two delivery paths for compressed and divided gas, its structure is shown in Fig 4, the arrows show the direction of airflow. The reducer of rated flow rate 25 m^3^/h is as a prototype of stainless steel pressure relief device used to fix output pressure, and its input end is connected with 25 L nitrogen cylinder by the matching cylinder valve, while the output pressure will be fixed to 2.30 MPa through the transformation without manual adjust to reduce steps of operation and prevent misuse, then two output gas paths are formed after the tee connector, so this output pressure will be act on the front input of DN15 self-reliant pressure regulating device without additional energy. The pressure regulating device uses the energy of controlled medium as the power source to introduce the actuators by its gas feedback tube to control the spool position, the control threshold 1.80 MPa at the back output of pressure regulating device is used as the evaluation criteria and control objective for continuous gas delivery, the pressure inside the cylinder is stabilized at the control threshold by changing the flow between front input and back output of pressure regulating device.

#### Large-diameter quick connection and adjustable pressure holding release technology

In order to quickly build a pipeline system with the large flow rate liquid and reliably prevent medium leakage on the overall flowing path, it is necessary to implement the technical specifications of devices with flowing liquid, differential quick connection types, and adjustment of jet status. The matching of parameters such as discharge flow rate, pressure, and diameter of pipeline network system should be studied, and the structures with convenient connection and reliable sealing should be constructed between the adjacent pipe bodies by differentiation, while reasonably designing the flow channel structure at the end of pipeline network.

**Fig 5:**
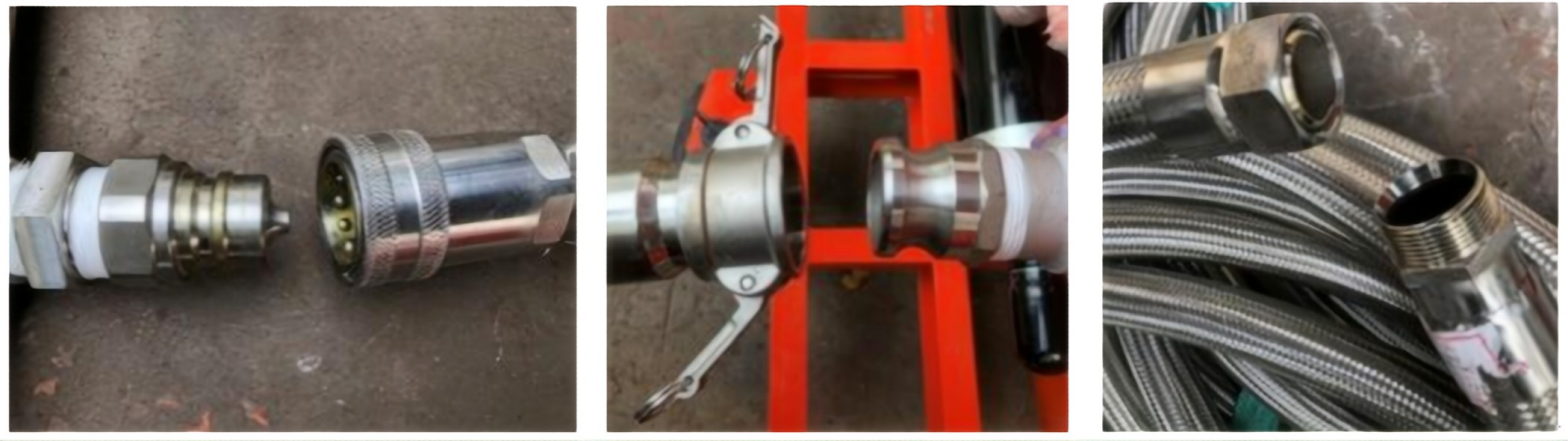
Quick connection type selected for liquid confluence and delivery. (a) Self-closing type, (b) Wrench type, (c) Arc surface seal type.

There are multiple sections of metal hoses with different lengths used for drainage of each insulated storage tank by the wrench type quick connectors, and which are collected by the self-closing quick connectors into confluence mean. In addition, the front end of combined fluid delivery pipe of 20 m each is connected to a valve at the outlet of the confluence mean by a wrench type quick connector, while the other end is connected in series with a control valve. The quick connectors and confluence mean are used for continuous drainage and centralized control, and the additional valve is set to the end of the delivery pipe to operate conveniently and reasonably. The three types of quick connection for liquid confluence and delivery are shown in Fig 5.

According to the highest working pressure 2.20 MPa when discharging liquid and the liquid release time (≤12 minutes) of a single insulation storage tank, the theoretical diameter of drainage pipe network made of stainless steel is calculated as no less than DN34. Therefore, its actual diameter must be designed as DN40, because the closest standard valve size is DN40.

Based on the physical characteristic of carbon dioxide, effective control and adjustment for injection angle and pressure as well as flow rate can be achieved with the aid of a nozzle, and the effect of gasification jet can be further improved. According to the indicators of effective storage volume 2 m^3^ per a single insulation storage tank, such as filling coefficient (≥0.95), liquid release rate (≥99%), liquid release time (≤12 min), the discharge rate is calculated as 165 L/min. A mechanical stainless steel nozzle of universal ball joint with nominal pressure 4.00 MPa and flow rate 176 L/min as well as maximum adjustable jet angle of 90° is selected. In addition, non-adjustable and straight through gasification nozzles of large-inside diameter are customized for easy matching, the serialized specifications are 16 mm, 21.5 mm and 28 mm.

#### Multiple safety protection and reliable transportation technology

When going deeper into disaster environments such as underground gas explosions, fires, and roof falls, it is necessary to address the issues of large tonnage integrated loading, combined coupling traction, and balanced rolling on narrow track. It is also necessary to address the issue of adopting vibration damping and collision prevention measures during movement to effectively avoid safety hazards caused by impact damage. A hanging structure of heavy load transportation suitable for underground track, and an anti-collision structure for external modules of tank body, as well as a method for effective attenuation of forced impact energy on the transmission path should be studied.

**Fig 6:**
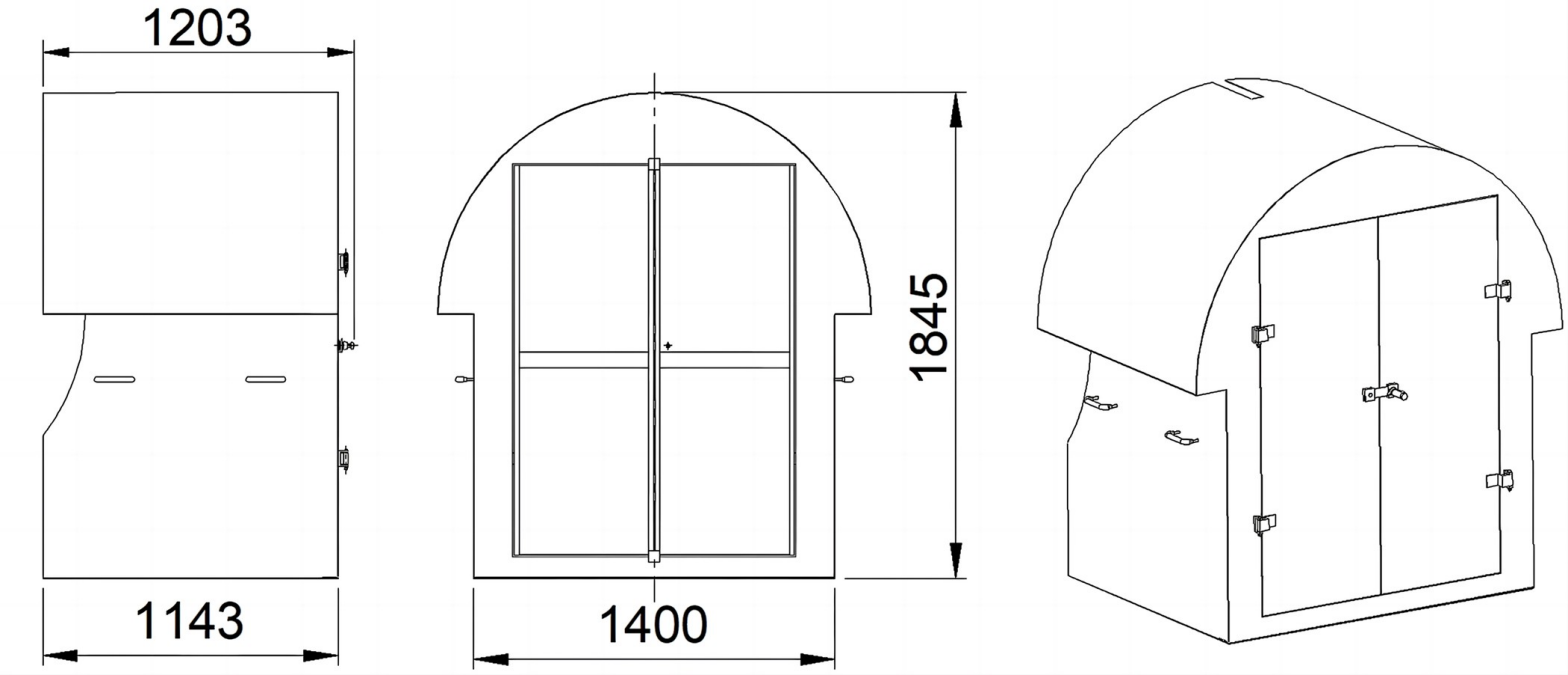
Structure diagram of protective shell.

In order to prevent the pipe valve system and self-pressurization control system from collision and damage in the complex environment, the following multiple safeguard measures are mainly adopted to resist or slow down shock:

1. The 3 mm thick steel plate is processed by cutting, coiling, bending and tailor-welding to form a closed protective shell to protect the pipe valve system, its structure is shown in Fig 6. When the pipe plugging and valve adjustment are needed, just open the double door on the front of protective shell;
2. Addition of vibration damping pads between the main components, and thicker design for outer layer of storage tank, as well as compact arrangement of pipe valve system.

**Fig 7:**
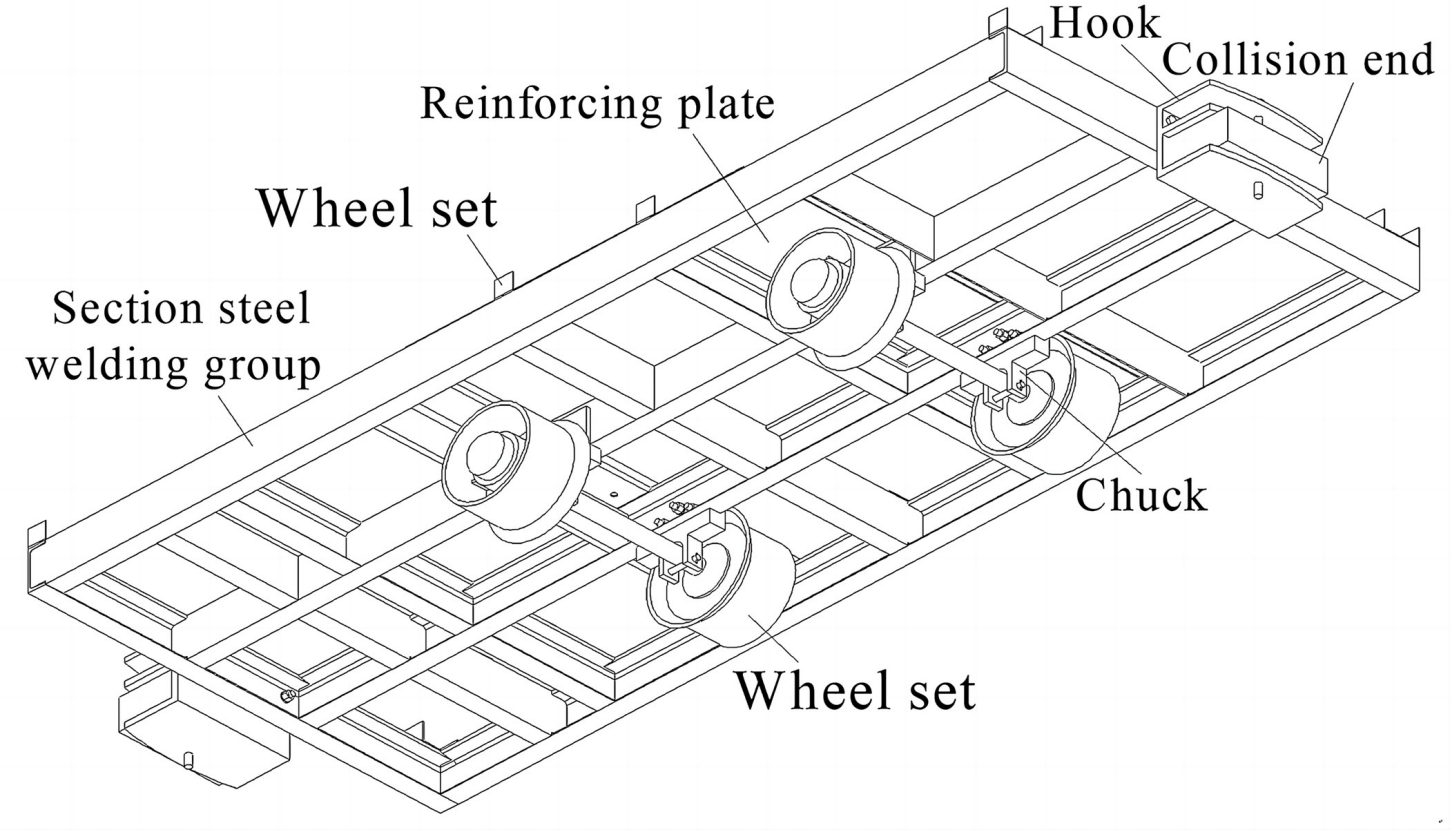
Structure diagram of mobile and hitching base.

Fig 7 shows the structure diagram of mobile and hitching base, which is composed of wheel set, hook, collision end, card slot, reinforcing plate and section steel welding group in truss form, each section steel welding group is fixedly connected with a storage tank and other main components by bolts. Two sets of wheels are arranged symmetrically on both sides of concentrated load centerline of storage tank on the base, and which are sleeved on the welding group through the chuck. According to the total weight 6 t of a single section after the insulation storage tank is filled, the solid special wheel set with the diameter of 350 mm is selected to ensure the carrying capacity, and the special wheel spacing of 600 mm or 900 mm is adopted to be suitable for narrow track transportation in Chinese mines.

### Equipment composition

This paper develops a new type of mobile equipment injecting the liquid carbon dioxide directly, which is suitable for accident emergency rescue of mine fire, gas explosion, coal and gas outburst, and can also be used to prevent spontaneous combustion in the coal mine goaf. The functions including perfusion, storage, transportation, liquid confluence, 100 m delivery and reliable gasification are gathered in integral whole. It is combined to form from five sets of mobile and articulated base with three-ring chains, where each base carries an insulation storage tank and a self-pressurized regulating system as well as a protective shell. A set of confluence system is installed at the front of first section, and the fluid delivery pipe of 100 m is matched to the outlet of confluence system. In order to adapt for narrow transportation space under the mine, the overall appearance of single section is controlled at 3850 mm (length)×1500 mm (width)×1950 mm (height).

**Fig 8:**
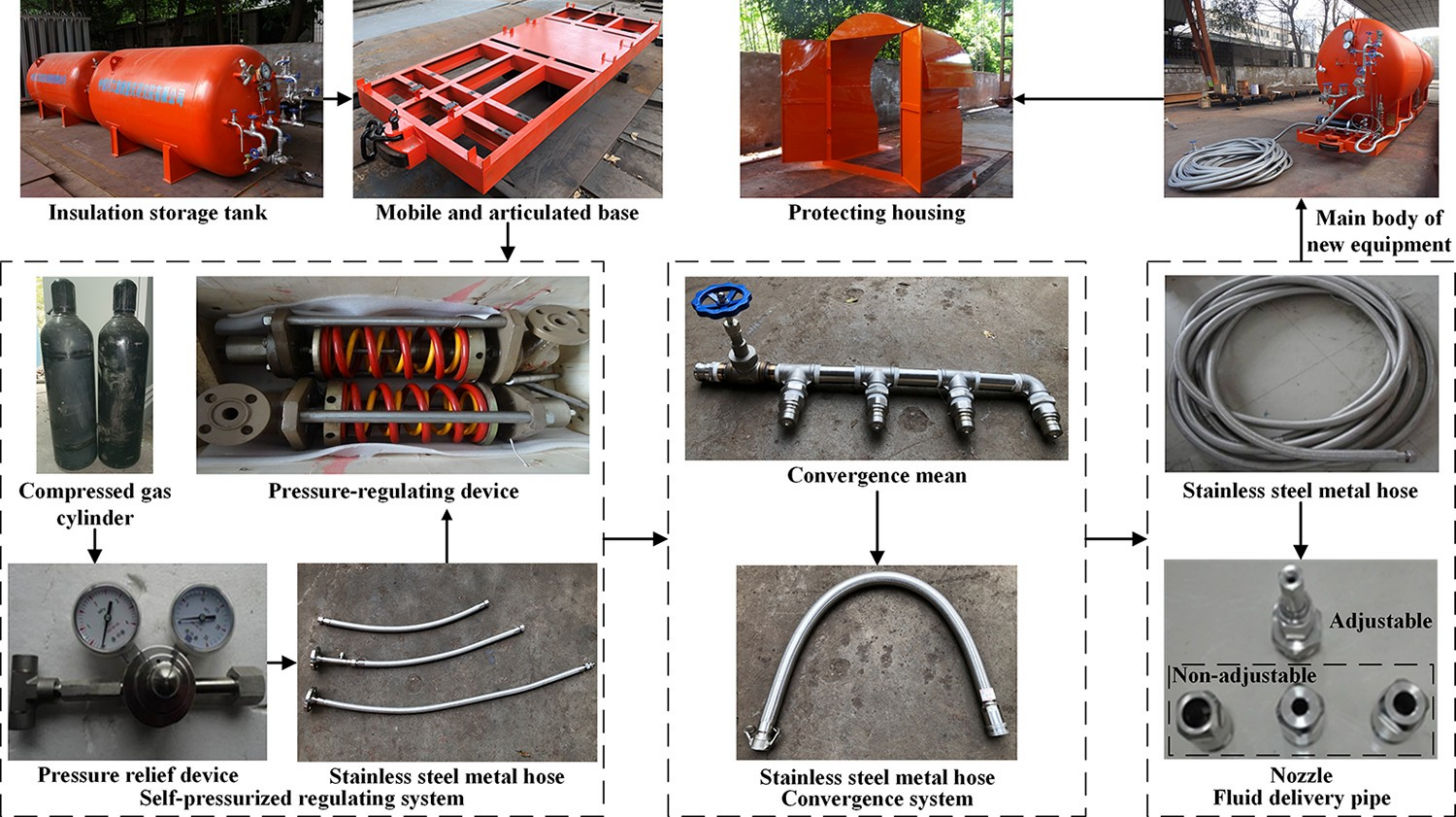
Combination of key components.

The combination of key components is shown in Fig 8 to reflect their system configuration and installation order clearly. When conditions of mine roadway construction and rail transportation are superior, the protective shell as accessory will be unequipped to reduce overall weight.

### Evaluation indicators

The performance influencing factors are directly related to the effect of new mobile approach in fire prevention and extinguishing or toxic gases diluting, which are used to evaluate the characteristic of liquid carbon dioxide inerting equipment. Each performance influencing factor needs to be tested and evaluated one by one, including persistence of low-temperature insulation storage, reliability of liquid pressure holding delivery, and adaptability and operability in the harsh environment. The performance influencing factors of new mobile approach is analyzed as follows:

1. For storage tank with compound type of vacuum interlayer and heat insulating powder, the vacuum degree and air leakage rate after vacuum extraction as well as daily evaporation rate are low to reduce the filling and maintenance costs, which represent insulation storage level after filling the liquid carbon dioxide. However, the leaks exist in the welds of any insulation storage tank, the number and size will directly affect the gas content that continuously enters the vacuum interlayer during long term use. Therefore, the welding condition is the decisive factor affecting the three, while the external environment temperature and use time are the objective factors affecting the vacuum degree and daily evaporation rate;
2. During the draining process, the pressure in the insulation storage tank and the entire drainage pipe network will gradually decrease. When the pressure is lower than 1.00 MPa, the liquid will instantly solidify and cause ice blockage, while the lowest pressure value of entire drainage pipe network appears at the end of drainage pipe, the pressure at this place should be the basis for measuring whether ice blockage occurs, which is affected by parameters such as medium characteristics, pipe specifications and along-path loss. If the pressure drop is small, the pressure gauge indication of tank can be used to predict the ice blockage. Therefore, the ability to resist pressure drop can be reflected by continuous discharge factor, and it is necessary to pressurize the tank to increase the initial pressure of discharge pipe to achieve pressure holding delivery;
3. When the liquid carbon dioxide releases at the end of liquid discharge pipe directly, the effect of extinguishing the fires or diluting the toxic gases is affected by its inerting characteristic, which is mainly reflected by the three factors of gasification jet and endothermic cooling as well as flame retardant. The former reflects the difference in the range of action when regulating the output of different flow rates, and also reflects the instantaneity of liquid-gas conversion, while the latter two reflect the effectiveness of inert medium to quickly cover the hazardous area, including reducing the concentration of combustion supporting or flammable gas components, and lowering the temperature.

## Experimental setup

### Experimental environment

The performance influencing factors were tested and analyzed under ambient environmental conditions in the temperature range of 5°C~33°C, and with the atmospheric pressure 101 kPa and relative humidity of 75%.

### Experimental principle

#### Vacuum degree

The vacuum detection mainly uses the changes in certain physical effects of gases at different pressures to measure the pressure. At present, the thermocouple vacuum gauge that uses the gas dynamics effect is widely used. In accordance with the Chinese standard ‘GB/T 18443–2010 Performance test method of vacuum insulated cryogenic equipment’, the formula for calculating the air leakage rate *Q* of interlayer by the method of vacuum detection is defined as follows.


$$Q=\frac{(p_{v2}-p_{v1})V_{i}K_{i}}{t}$$ (12)

Here, *p*_*v*2_, *p*_*v*1_ denote the vacuum degree of interlayer tested twice respectively; *v*_*i*_ denotes the interlayer volume, it takes 2.89 m^3^; *K*_*i*_ denotes the coefficient, it takes 0.6 for vacuum powder insulated container; *t* denotes the resting time.

#### Insulation storage

In the method of calculating the daily evaporation rate for LNG storage tank, the method of liquid level difference is most suitable for long term monitoring of liquid volume in the tank, which is based on the actual change of effective liquid volume after 24 hours to convert the daily evaporation loss. The formula for calculating the daily evaporation rate *α*_1_ by this method is defined as follows [30].


$$\alpha_{1}=\frac{P_{1}(V_{1}‐V_{2}\mathrm{)(}T_{1}‐T_{2})}{nV_{3}P_{2}(T_{3}‐T_{2})}\varphi \mathrm{\times 100\%}$$ (13)

Here, *V*_1_, *V*_2_, *V*_3_ denote the initial, final and effective liquid volume respectively; *φ* denotes the calibration factor of level instrument; *n* denotes the number of days measured; *P*_1_, *P*_2_ denote the average atmospheric pressure and atmospheric pressure in the standard state respectively; *T*_1_, *T*_2_, *T*_3_ denote the reference temperature, boiling temperature of test medium and actual measured value of ambient temperature respectively. When calculating the daily evaporation rate of LNG storage tank using the method of liquid level difference, the influence of atmospheric pressure can be ignored, so this has little effect on the calculation results because *P*_1_ and *P*_2_ are approximately equal.

In addition, the ambient temperature change was huge throughout the test cycle, but the average was not less than the reference temperature 20°C. Therefore, the Formula (13) can be simplified based on Formula (14) as following Formula (15).


$$T_{3}‐T_{2}\geq T_{1}‐T_{2}$$ (14)

$$\alpha_{2}=\frac{V_{1}‐V_{2}}{nV_{3}}\mathrm{\times 100\%}$$ (15)

Here, *α*_2_ denotes the simplified daily evaporation rate of tank, which should not be less than *α*_1_ accurately calculated by Formula (13). Therefore, *α*_2_ calculated from Eq (15) has a theoretical basis as the daily evaporation rate of liquid carbon dioxide inerting equipment, the test results will tend to be conservative.

#### Pressure holding delivery

**Fig 9:**
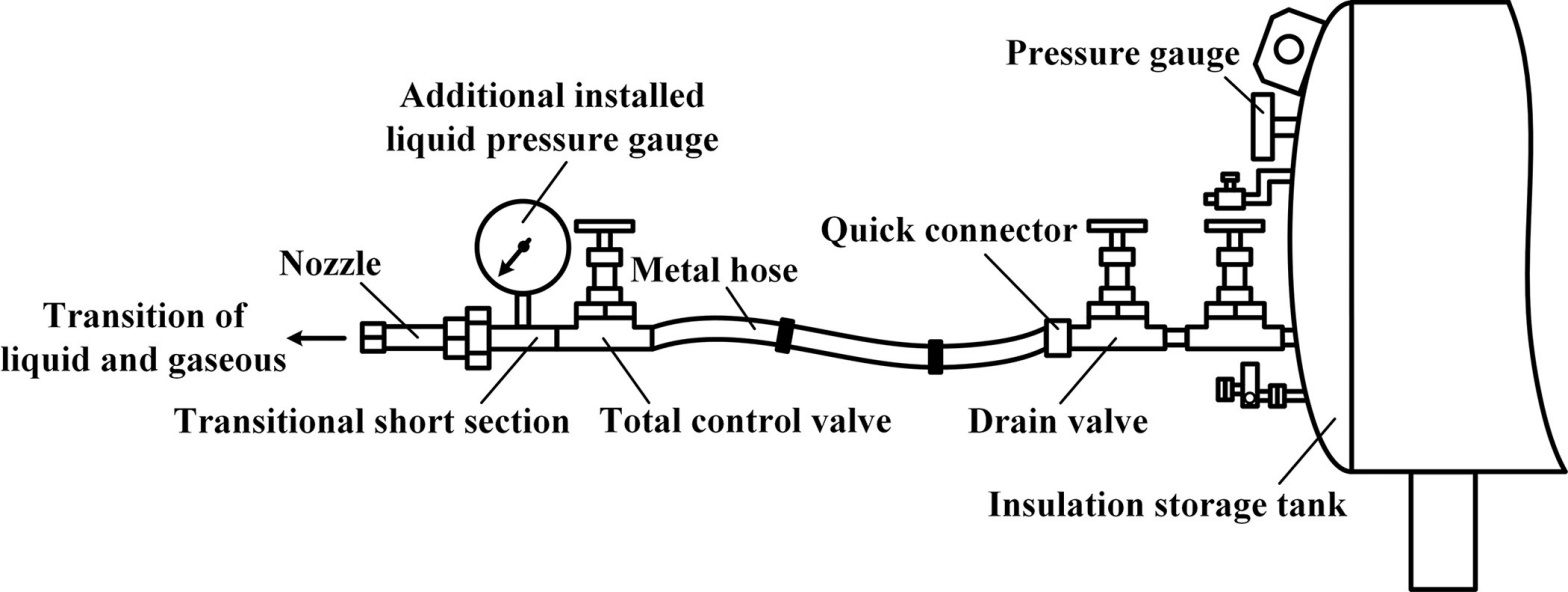
Installation of liquid pressure gauge at pipe end.

The pressure gauge matched with the tank is used to monitor the gas pressure in the upper part of tank in real time. Due to various factors such as loss along the pipe and local loss of pipe inlet as well as valve pressure drop, it is necessary to connect the transitional short section at the front end of nozzle and install a liquid pressure gauge for test analysis whether there is a big difference between the liquid jet pressure delivered to the end of 100m pipe and the indicated value of tank pressure gauge, the specific installation situation is shown in Fig 9.

#### Gasification jet

Due to the sudden change of ambient temperature and pressure, the conversion of carbon dioxide from liquid to gas will be realized [31]. This cryogenic liquid is discharged from the closed pipe to the outside space, especially the high temperature fire zone, the temperature difference between the inside and the outside of pipe is large, and the pressure at the end of pipe is reduced to 1 atmosphere, so it can instantly vaporize and expand in a jet state due to the release of pressure.

#### Endothermic cooling

**Fig 10:**
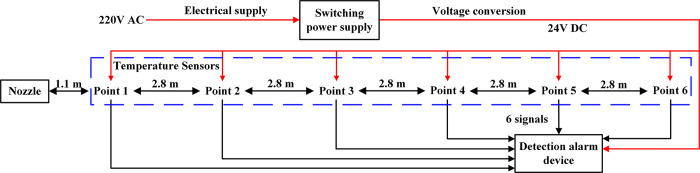
Selection and layout of temperature test equipment.

Gas molecules have higher internal energy than liquid molecules, so when liquid molecules become gas molecules, they need to absorb heat from the outside to reduce the ambient temperature. As shown in Fig 10, the adjustable nozzle is install fixedly to jet the liquid in the horizontal direction, and a temperature sensor at 1.2 m from the ground is set as measuring point 1 at a distance of 1.1m from the nozzle in the horizontal direction, then taking this measuring point as the starting point, five temperature sensors are arranged in sequence to form measuring points 2, 3, 4, 5, 6 along the center line of cone jet range at 2.8 m intervals. The current signals output by the thermal resistance temperature sensors are connected to the multi-channel detection alarm device to realize the conversion and display of digital signals via wired communication.

#### Flame retardant

The flame retardant using carbon dioxide is to reduce the concentration of oxygen [32, 33] and combustible gas components in the air in hazardous area, so that the fire source loses the burning conditions. On the moment when the fire source extinguished, the critical oxygen concentration of wood and gasoline shall not exceed 16.98%, and the concentration of carbon dioxide when injecting carbon dioxide into the fire zone for inerting shall not be less than 28.6% [31].

#### Continuous discharge

The insulation storage tank is usually filled with the pressure of up to maximum value 2.20 MPa, and discharge starts under full liquid, and the pressure drop in the tank is small in the early stage. When the pressure in the tank is lower than the pressure regulator settings, it can be stabilized through the replenishment of nitrogen bottle for a long time. However, the state of pressure retention at the end of discharge will be broken, and the supplemental air volume cannot make up for pressure loss. The continuous discharge depended mainly on the remaining gas volume of nitrogen bottle and the compensation rate, which can effectively slow down the pressure drop until the liquid is completely discharged.

### Experimental procedure

**Fig 11:**
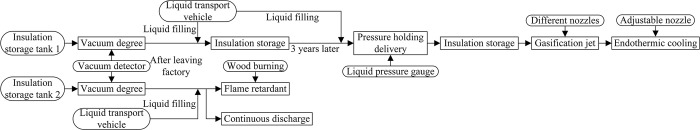
Experimental procedure of performance influencing factors.

**Fig 12:**
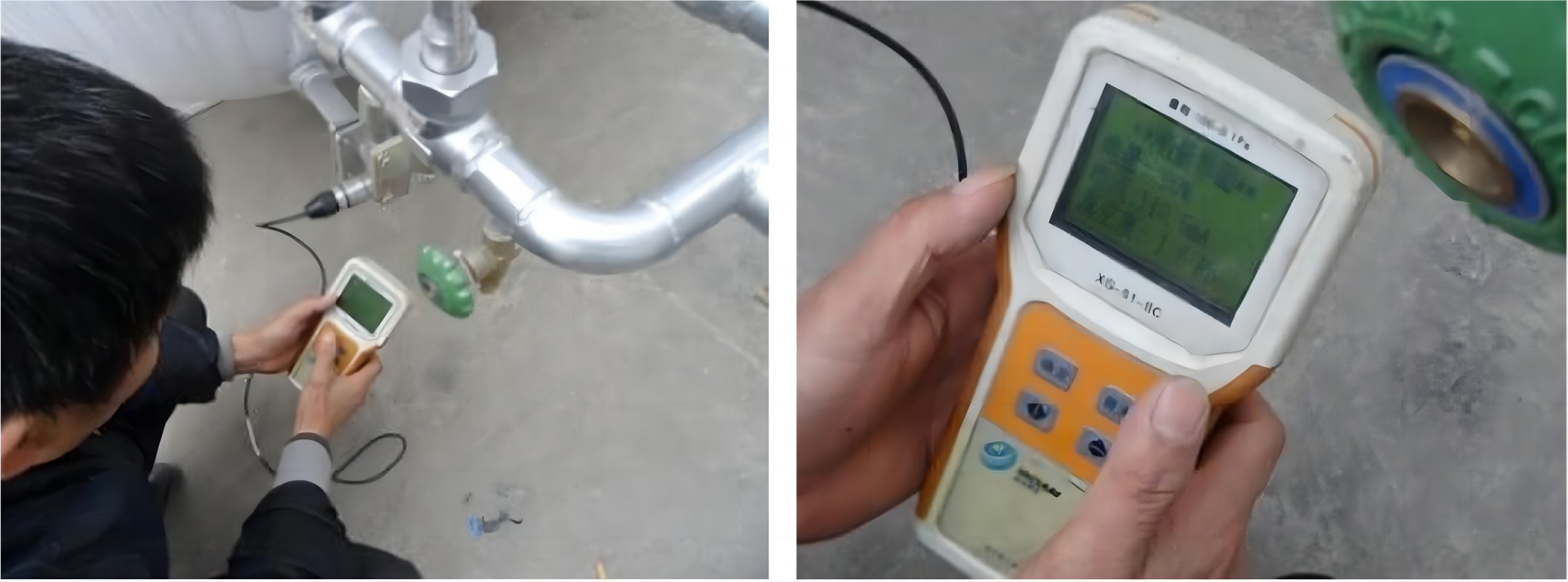
Vacuum detection.

**Fig 13:**
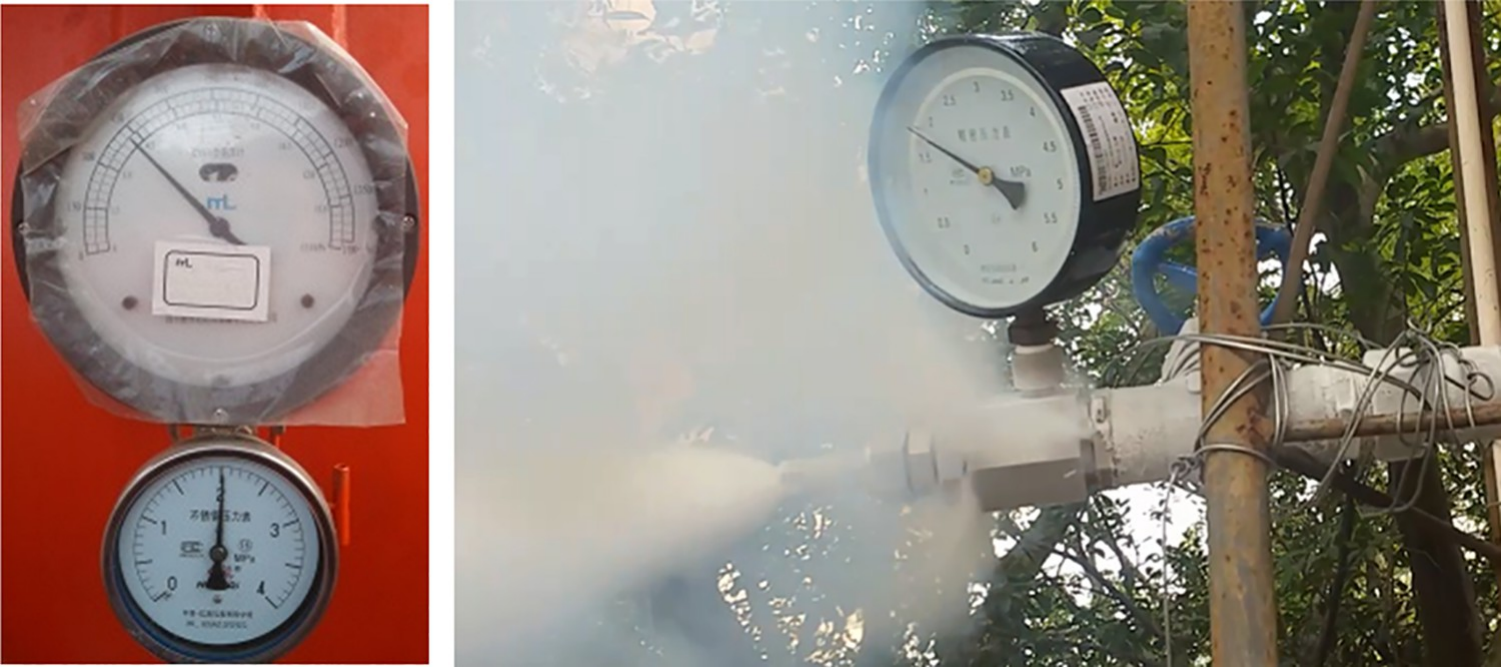
Comparison of pressure gauge readings. (a) Pressure gauge reading of tank, (b) Pressure gauge reading at pipe end.

**Fig 14:**
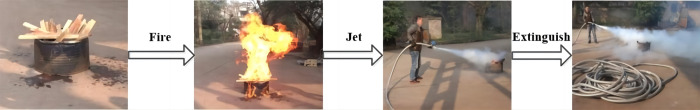
Effective disposal process of simulated fire source.

The experimental procedure of performance influencing factors as shown in Fig 11, selected two insulation storage tanks as the object to be tested as follows:

(1) Carried out an experiment of vacuum detection for two tanks before leaving the factory, then tank 1 filled with liquid used to test insulation storage factor after the factory.
1) The protective cover on the metal thermocouple gauge as vacuumize connection device was unscrewed, and the signal line of the hand-held vacuum detector was plugged in to measure the vacuum degree in the interlayer, then measured the vacuum degree again after 5 days to calculate the air leakage rate of the interlayer. The vacuum detection situation is shown in as shown in Fig 12;
2) The tank 1 filled with liquid was put on for a long time with regular observation and record of readings for pressure gauge and level gauge, thus the technical indicator of daily evaporation rate was calculated. The experiment was divided into two groups, where the data of 12 times was recorded by the same time interval respectively, then when the same holding tank was put into use for 3 years, the liquid volume and pressure in the tank was recorded at an interval of once a day, so the daily evaporation rate would be recalculated.
(2) The laying of 100 m pipe was completed, one end was connected to the discharge valve of tank 1 or 2 filled with liquid, and the other end is connected to the total control valve of discharge and adjustable nozzle. On the basis of satisfying the following five experimental conditions in sequence, opened the discharge valve and total control valve in turn to discharge liquid in the tank, then the valve of nitrogen bottle and gas booster valve Ⅱ were opened to put the self-pressurization control system in automatic control mode.
1) Installed the liquid pressure gauge between the adjustable nozzle and the total control valve according to Fig 9, and recorded pressure gauge readings from the inside of tank 1 and the pipe end after stable discharge to test the pressure holding delivery factor. The comparison of pressure gauge readings from the inside of tank and pipe end is shown in Fig 13;
2) For tank 1, replaced the different specifications of gasification nozzles in turn, and photographed its jet situation respectively from the same perspective under the same working pressure to test the gasification jet factor;
3) For tank 1, fixed the adjustable nozzle and arranged the temperature test equipment according to Fig 10, then recorded the temperature values of each measuring point from the beginning of gasification jet to the steady state at 10 s intervals to test the endothermic cooling factor;
4) For tank 2, the wood poured with gasoline was filled in drum and ignited, then pointed the adjustable nozzle at the fire source when the wood was burning steadily to test the flame retardant factor, the process is shown in Fig 14;
5) The later stage parameters from the normal discharge of tank 2 were detailed and analyzed in detail. In this partial process, the liquid volume and pressure in the tank as well as the pressure in the nitrogen cylinder were recorded every 0.5 min by observing the indicators to test the continuous discharge factor.

### Assumption list

#### Vacuum degree

The vacuum pumping operation of insulation storage tank interlayer is completed in a low humidity environment outside the tank, and the vacuum degree test value is extracted at the lowest pressure state inside the interlayer. In addition, the time span of two vacuum degree tests on the same insulation storage tank is sufficient, so the welding defects of insulation structure can be fully exposed, then the calculated interlayer leakage rate based should be able to intuitively reflect the dynamic airtightness of interlayer.

#### Insulation storage

During the process of holding liquid in an insulated storage tank, the temperature outside the tank is not constant due to weather changes, so the average temperature value is taken as the environmental condition to measure the daily evaporation rate level.

#### Pressure holding delivery

A combination pipeline with a length of 100 m has all connection points sealed without leakage, and the distance between the liquid pressure gauge and the adjustable nozzle end is set reasonably, so the pressure detection position is just outside the mixed flow area inside and outside the pipeline, which can truly reflect the liquid delivery pressure at the pipeline end.

#### Gasification jet

The injection time of different nozzles is extremely short, and the small amount of liquid consumption does not cause a significant change in the pressure inside the tank, resulting in the injection pressure of each nozzle being close.

#### Endothermic cooling

The maximum wind speed during the liquid injection process does not exceed level 1 (0.3–1.5m/s), causing the jet airflow to complete the physical state transition and generate refrigeration efficiency, without being affected by environmental airflow.

#### Flame retardant

The maximum wind speed during the liquid injection process does not exceed level 1 (0.3–1.5m/s), resulting in stable environmental airflow that does not affect the combustion and extinguishing of fire source.

#### Continuous discharge

During the process of discharging in a full liquid state, there are a few brief pauses, but during which a slow increase in pressure and evaporation loss of liquid stored in the tank are not significantly caused.

## Results and discussion

### Vacuum degree

The initial air pressure in the vacuum interlayer of tank 1 and 2 was read as 1.5 Pa and 1.6 Pa respectively by the hand-held vacuum detector, which were significantly lower than the maximum value 2.0 Pa specified in the Chinese standard ‘GB/T 18442–2019 Fixed vacuum insulated cryogenic pressure vessel’. The vacuum test values of tank 1 and 2 after being left for 8 days were both 1.8 Pa, then calculated the air leakage rate of tank 1 and 2 according to Formula (12) to be 7.5×10^−7^ Pa·m^3^/s and 5.0×10^−7^ Pa·m^3^/s respectively, which were much smaller than the maximum value 1.0×10^−5^ Pa·m^3^/s specified in the previous standard. Although calculated based on this air leakage rate, it only takes 13.4 days and 16.1 days for the interlayer vacuum degree to reach 2.0 Pa, the dynamic change of interlayer vacuum degree with time is nonlinear, and the actual time will far exceed the theoretical calculation value. The tank 1 and 2 after leaving the factory, the vacuum degree should be inspected every 6 months or so, and the vacuum should be pumped every 5 years on average.

### Insulation storage

**Fig 15:**
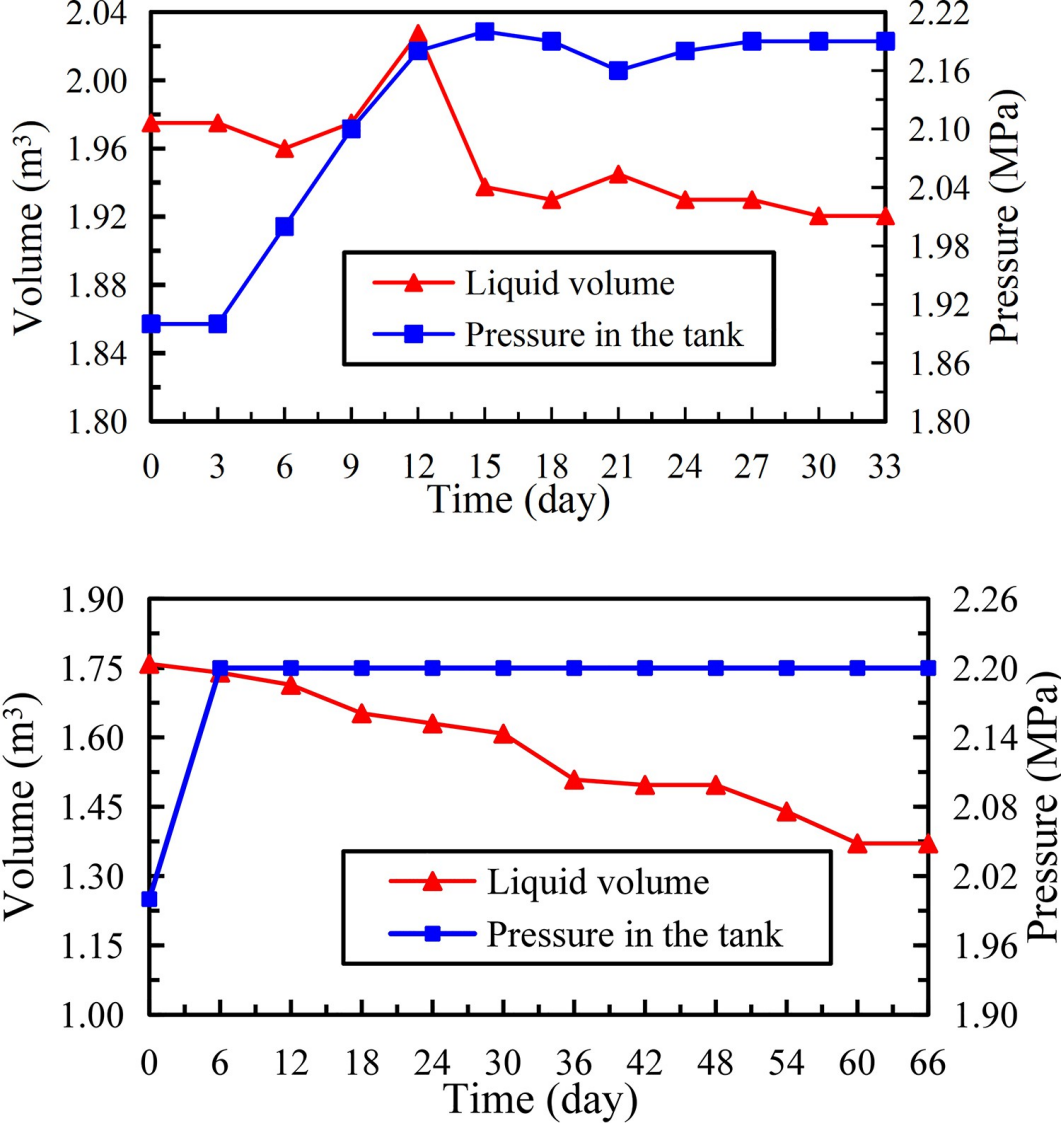
Change of liquid volume and pressure in the tank. (a) Group 1 test, (b) Group 2 test.

The working conditions for the two groups were: (1) Ambient temperature was in 5°C~25°C with average value of about 20°C, and the cycle was 33 days. (2) Ambient temperature was in 15°C~33°C, where the temperature exceeded 20°C for most of the time, and the cycle was 66 days. The test data trends of insulation storage performance are shown in Fig 15 (S1 Data).

According to the correspondence between the liquid height and volume of level gauge, the liquid volume was calculated by converting the level gauge readings to determine the daily evaporation rate. For the two groups of experiments, the pressures in the tank showed an overall upward trend with time, while the change trends of liquid volume in the tank were opposite, so this change characteristic combines the dynamic transformation law of liquid and gas. At the beginning of group 1 test, the liquid in the tank was full, and then decreased from 1.97 m^3^ (filling coefficient 98.5%) to 1.92 m^3^ with a total reduction of 0.05 m^3^, so the daily evaporation rate was 0.08% approximately. During most of group 2 test, the ambient temperature was above 27°C, the liquid volume was reduced from 1.76 m^3^ to 1.37 m^3^ with a total reduction of 0.39 m^3^, where the daily evaporation rate was not more than 0.30%. The higher the outside temperature, the faster the gasification rate of upper liquid in the inner cylinder, and the greater the volume of gas discharged through the automatic pressure relief device. Although the high ambient temperature caused the daily evaporation rate of second group to be higher than the first group, it still did not exceed national standard 0.30% (under 20°C and 0.10 MPa absolute pressure). Taking into account the extreme situation, according to the daily evaporation rate of 0.30%, the effective liquid volume in the tank would be about 1.69 m^3^ after 48 days of full liquid storage.

**Fig 16:**
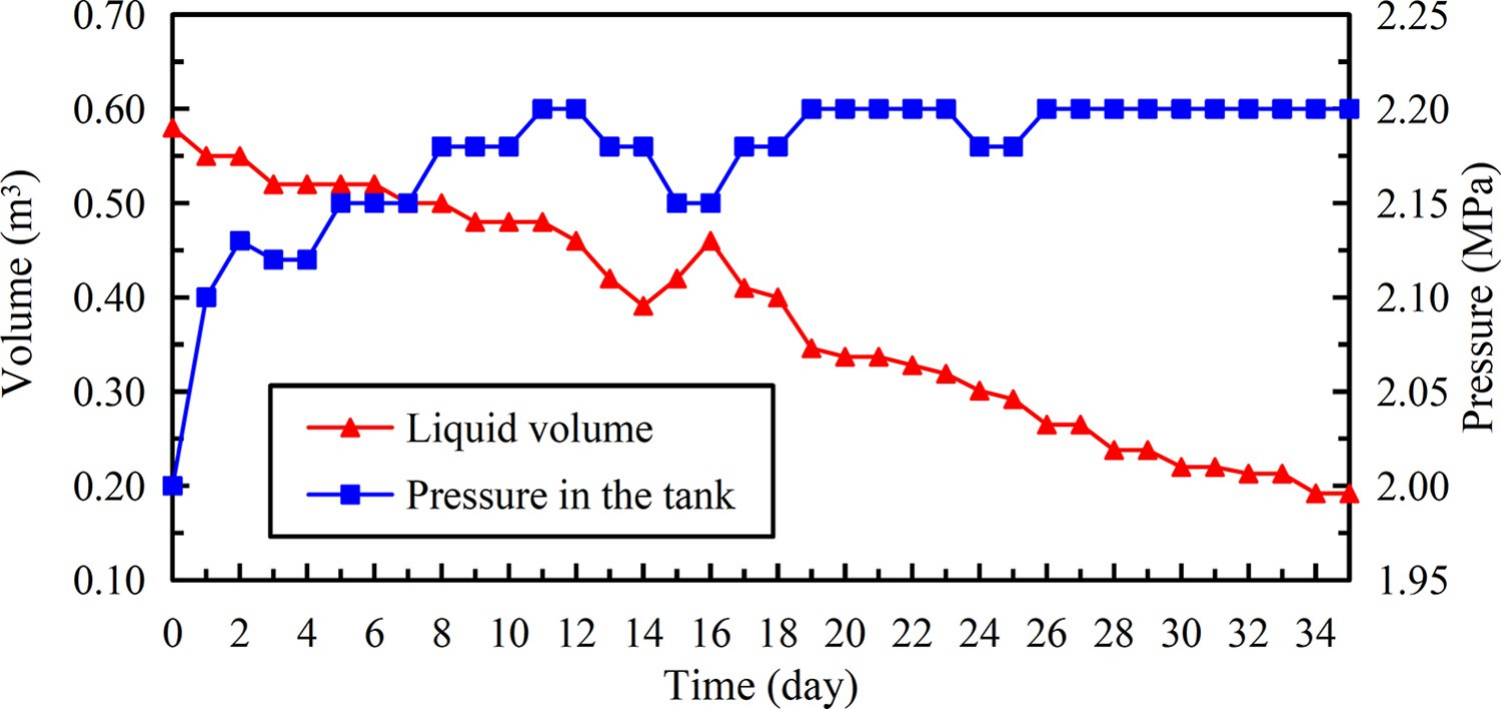
Change of liquid volume and pressure in the tank put into use for 3 years.

The data of insulation storage performance under 15°C to 32°C for 35 days after 3 years are shown in Fig 16 (S1 Data). The liquid in this test decreased from 0.58 m^3^ to 0.192 m^3^ with a total reduction of 0.388 m^3^, and the daily evaporation rate was about 0.55%. It was 1.8 times the corresponding value of second group test, and the effective liquid volume in the single tank of full liquid storage is not less than 1.44 m^3^ after 48 days.

### Pressure holding delivery

**Fig 17:**
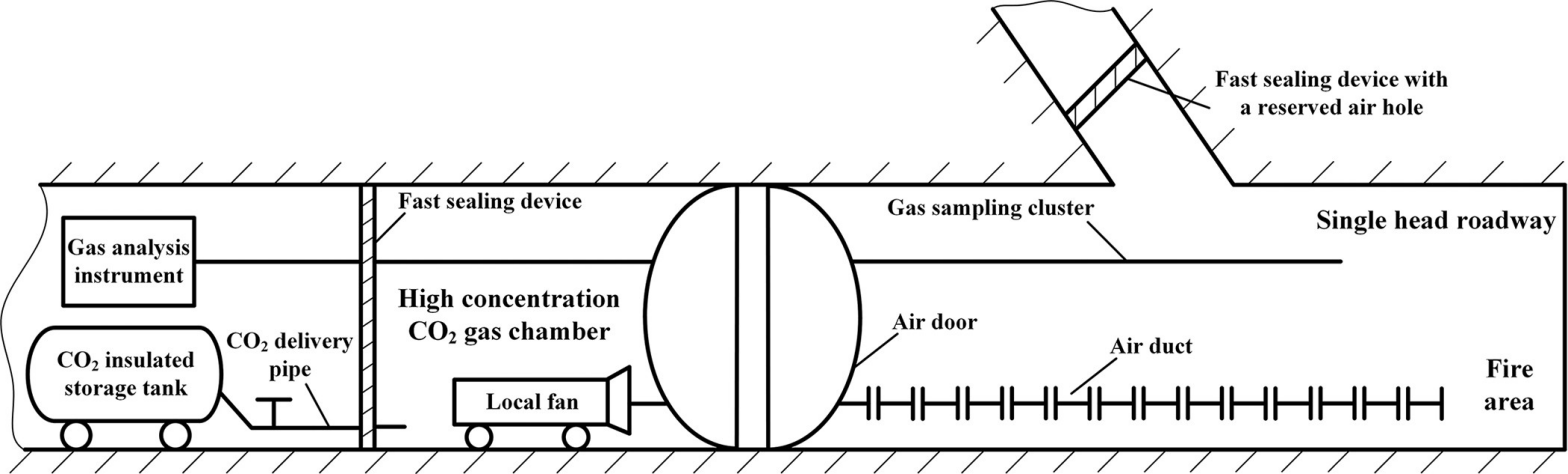
Technical principle for extending inerting distance of liquid carbon dioxide.

The pressure gauge reading of tank before and during the discharge was about 2.05 MPa, and the pressure gauge reading at the end of pipe was about 2.00 MPa, indicating that the two values were basically the same. Therefore, the various pressure losses caused by the pipe length of 100 m, the roughness of pipe inner surface and the mated valves are negligible. The actual working pressure at the outlet of nozzle can be grasped by observing the pressure gauge matched with the tank in the gasification injection process. During the process of carbon dioxide liquid flowing in the pipe, the pressure gradually decreases, and the temperature difference between the inside and outside of pipe makes it easy for the liquid to gradually vaporize and lose its cooling effect. The maximum pipe length currently does not exceed 200 m, but the underground sealing device is usually built safely away from fire zones or toxic gas accumulation sites, making it difficult to ensure that the liquid can be prevented from ice blockage and gasification after extending the delivery distance, and effectively injecting into the inert zone to achieve dilution and cooling functions. Further research is carried out on the feasibility of long distance delivery for inert medium under the well, and the technical plan is formulated:

1. On the basis of adding liquid pressurizing equipment at the front end of pipe, the heat preservation technology for pipes of heating ventilation and air conditioning can be used to cover the outer surface of stainless steel metal hoses, so the liquid delivery pipe with an insulating layer can be further extended, and all or part of liquid vaporizing during the flow process will be avoided;
2. The mobile liquid carbon dioxide fire extinguishing device is used in conjunction with local fan, air duct, sensor and air door to transport the gasified carbon dioxide to the fire area along the long distance underground roadway to achieve rapid inerting. As shown in Fig 17, close a section of roadway at the place where the local fan is placed as a gas chamber, and inject liquid carbon dioxide into this gas chamber to form and store the high concentration gas, then the cooling gas produced by liquid vaporization is sent to the fire extinguishing site by the air duct after being inhaled through the suction port of local fan. The effect of carbon dioxide gas accumulation in the lower space will be ideal when the single head roadway is downhill, because its density is greater than that of air. If the roadway is flat or uphill, the roadway is closed with fast sealing device, and a reserved air hole is left in the upper part of fast sealing device to detect the gas concentration of return air flow, so the time of closing the vent hole and stopping the local fan is determined.

### Gasification jet

**Fig 18:**
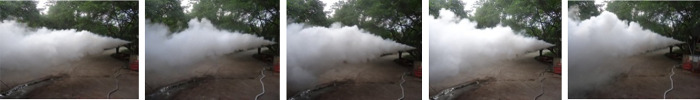
Comparison of jet conditions for different nozzles. (a) Adjustable, (b) 16 mm in diameter, (c) 21.5 mm in diameter, (d) 28 mm diameter, (e) No any nozzle.

Fig 18 shows the jet situation of different gasification nozzles when the positions of camera and nozzle were fixed. The mixture of liquid and gas were jetted into the continuous waves of white mist, the visual length range was not less than 15 m, and the maximum diameter was not less than 5 m, and the amplitude and distance gradually expanded with the increase of nozzle inside diameter.

### Endothermic cooling

**Fig 19:**
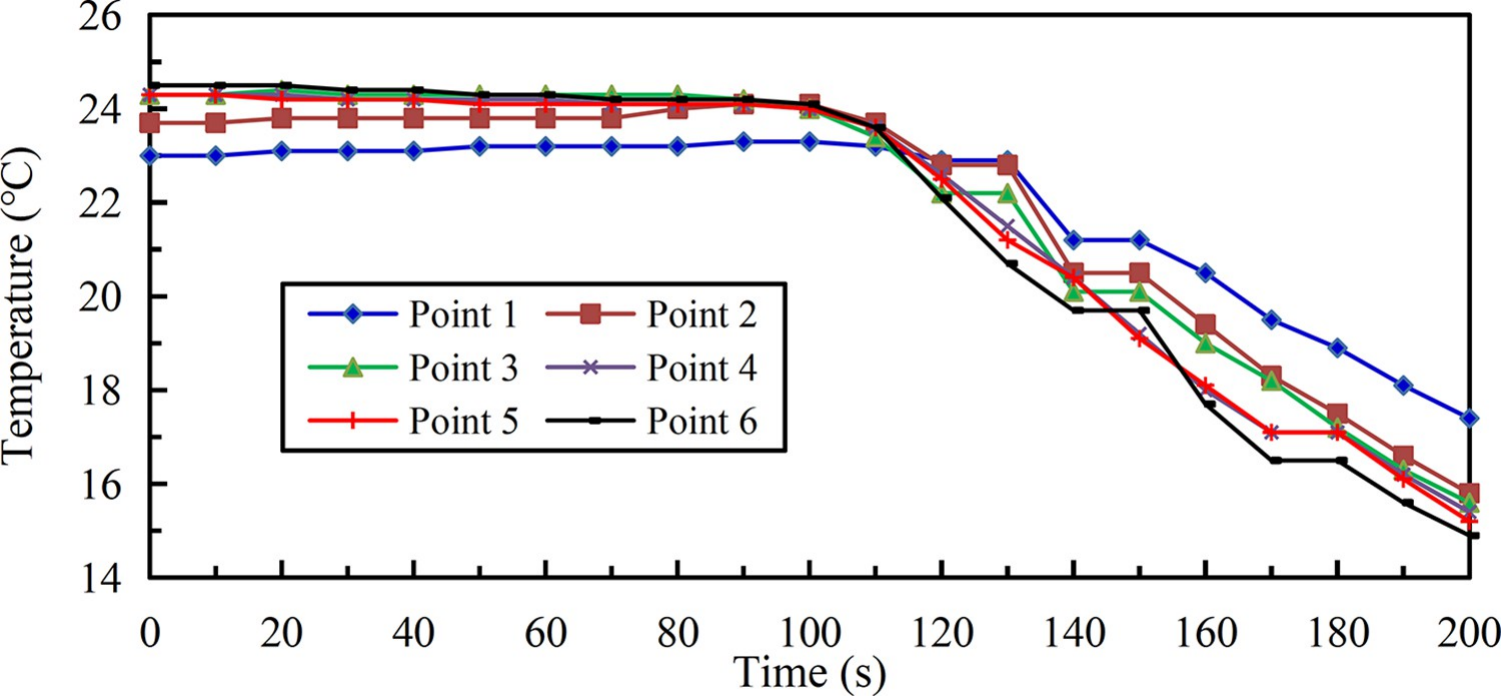
Change curves of temperature in 200 s on liquid gasification jet path.

The difference from endothermic cooling effect at each measuring point after liquid gasification jet under 2.20 MPa is shown in Fig 19 (S1 Data), the following characteristics in 200 s can be summarized as: (1) When the liquid was jetted, the temperature of each measuring point decreased significantly with time, but in the initial stage after the liquid in the pipe entered the external environment, there was a process of gradually increasing the jet distance to steady vaporization and heat absorption, resulting in temperature would not drop immediately. The temperature at measuring points 4, 5, 6 farther from the nozzle would respond faster to the action of inert medium, the temperature at the most sensitive measuring point 5 began to drop at 20 s, while the measuring points 1, 2, and 3 were closer to the nozzle, but the liquid needed to be jetted for a certain distance under pressure, that is, the liquid needed to stay in the environment outside the pipeline for a short time in order to fully exchange heat to achieve complete gasification, so the temperature response at these three measuring points was slower. Among them, the temperature at the least sensitive measuring points 1 and 2 showed a downward trend at 110 s respectively; (2) When the temperature at each measuring point is in the falling stage, the measuring point farther away from the nozzle at the same time started endothermic cooling earlier, so its temperature was lower. The measuring point 6 had the lowest temperature of 14.9°C at 200 s, the maximum temperature change range reached 9.6°C; (3) Adopt polynomial fitting method to form the temperature calculation formula of each measuring point changing with time as follows.


$$\left\{ \begin{array}{c} y_{1}=2.28\times 10^{‐10}x^{5}‐9.5866\times 10^{‐8}x^{4}+1.1656546\times 10^{‐5}x^{3}‐5.03610343\times 10^{‐4}x^{2}\\ +9.590977002\times 10^{‐3}x+22.993904358601\\ y_{2}=2.27\times 10^{‐10}x^{5}‐7.882\times 10^{‐8}x^{4}+5.316818\times 10^{‐6}x^{3}+1.2259834\times 10^{‐4}x^{2}\\ ‐9.101912735\times 10^{‐3}x+23.773315382265\\ y_{3}=1.6\times 10^{‐11}x^{5}+2.2133\times 10^{‐8}x^{4}‐1.0551306\times 10^{‐5}x^{3}+1.011765596\times 10^{‐3}x^{2}\\ ‐2.6747634081\times 10^{‐2}x+24.413377928394\\ y_{4}=1.59\times 10^{‐10}x^{5}‐3.1262\times 10^{‐8}x^{4}‐4.784352\times 10^{‐6}x^{3}+8.79191702\times 10^{‐4}x^{2}\\ ‐3.2347051910\times 10^{‐2}x+24.449169962407\\ y_{5}=7.9\times 10^{‐11}x^{5}+6.924\times 10^{‐9}x^{4}‐1.1151212\times 10^{‐5}x^{3}+1.318020364\times 10^{‐3}x^{2}\\ ‐4.3609127327\times 10^{‐2}x+24.477274032247\\ y_{6}=9.4\times 10^{‐11}x^{5}+4.8\times 10^{‐10}x^{4}‐9.966727\times 10^{‐6}x^{3}+1.173731468\times 10^{‐3}x^{2}\\ ‐3.8298465054\times 10^{‐2}x+24.665188726212 \end{array}\right.$$ (16)

Here, *y*_1_, *y*_2_…, *y*_6_ denote the temperature of measuring points 1, 2…, 6 respectively; *x* denotes the time after liquid gasification jet. The characteristic quantities of degree of fit for each measuring point are shown in Table 2.

**Table 2:** Characteristic quantities of degree of fit for each measuring point.

Measuring point	1	2	3	4	5	6
Maximum deviation *ΔT*_*H*_/°C	0.6859	0.9204	0.8097	0.4551	0.6063	0.6272
Minimum deviation *ΔT*_*L*_/°C	0.0043	0.0009	0.0004	0.0080	0.0461	0.0158
Average deviation *ΔT*_*A*_/°C	0.1079	0.1797	0.1733	0.2203	0.2473	0.2896
Decisive coefficient *R*^2^	0.9894	0.9899	0.9930	0.9927	0.9919	0.9897

Here, the ratio *R*^2^ for regression sum of squares to total sum of squares is used to reflect the degree of fit between the estimated value of trend line and the corresponding actual data, also known as the decisive coefficient, and the larger the decisive coefficient between 0 and 1, the higher the degree of fit. Using polynomial fitting method, the degree of fit is mainly affected by the highest order and the decimal places of highest order coefficient. Since the decisive coefficients of temperature calculation formula group (16) is within 0.9894 and 0.9930, and the average temperature deviation for each measuring point before and after fitting does not exceed 0.2896°C, the fitting effect is ideal by the 5th order polynomial fitting and keeping 12 decimal places. After the above fitting method is performed on the average temperature of each measuring point at the same time, the formula for calculating the average temperature *y*_*m*_ over time with the decisive coefficient 0.9946 is formed as follows:

$$y_{m}=1.34\times 10^{‐10}x^{5}‐2.9402\times 10^{‐8}x^{4}‐3.246706\times {\mathrm{10}}^{‐6}x^{3}+6.66949521\times 10^{‐4}x^{2}$$

$$‐2.3418869018\times 10^{‐2}x+24.128705065019$$ (17)

On the basis of Formula (17), add the constant (*T*_0_-24.0) to *y*_*m*_, then the crude calculation formula of temperature *y*_*m*1_ changing with time on the center line of liquid gasification jet is obtained as follows:

$$y_{m1}=1.34\times 10^{‐10}x^{5}‐2.9402\times 10^{‐8}x^{4}‐3.246706\times 10^{‐6}x^{3}+6.66949521\times 10^{‐4}x^{2}$$

$$‐2.3418869018\times 10^{‐2}x+T_{0}+0.128705065019$$ (18)

**Fig 20:**
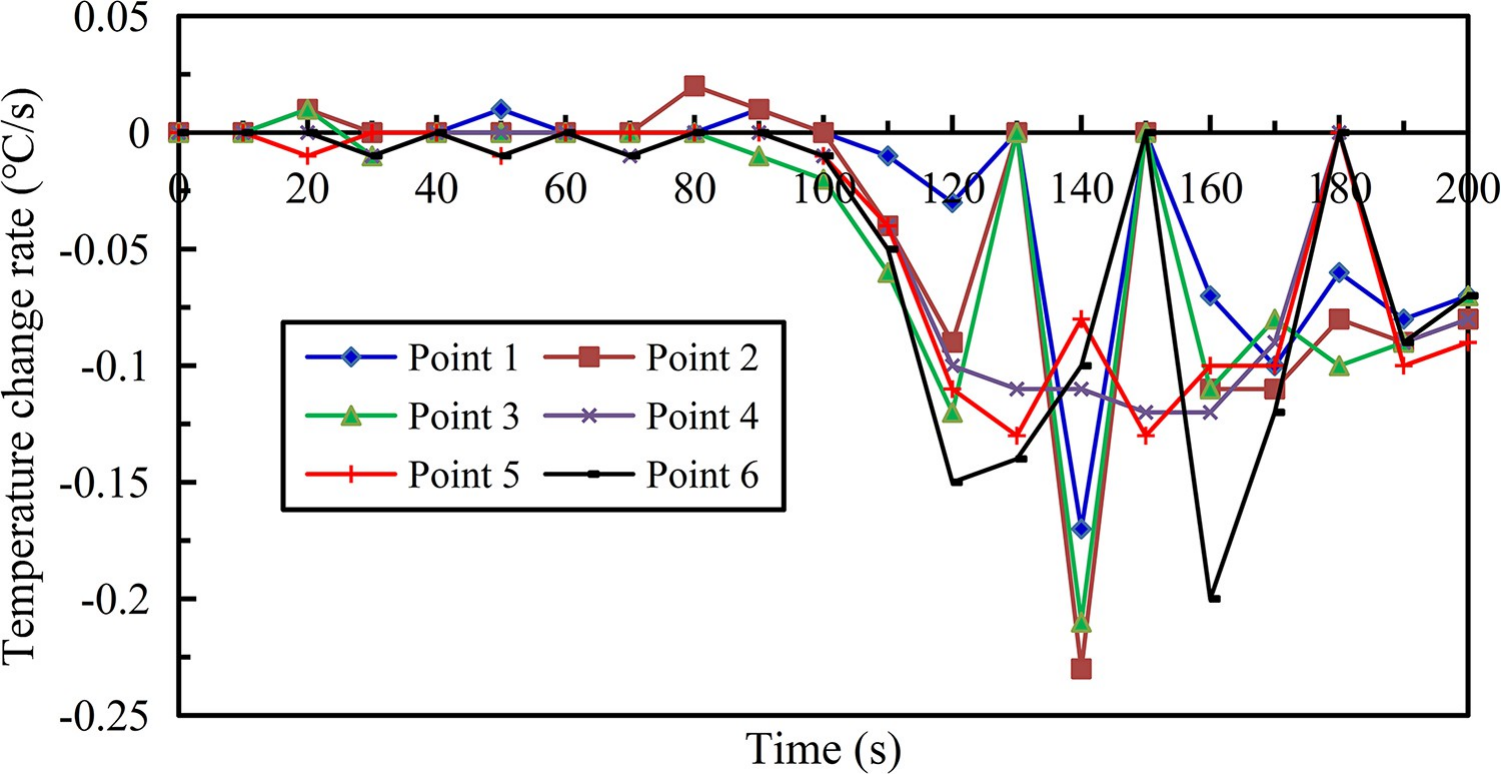
Temperature change rate curves in 200 s on liquid gasification jet path.

Here, *T*_0_ is the measured initial temperature before liquid injection, the average initial temperature before liquid injection is 24.0°C. The temperature change rate in 200 s on the liquid gasification jet path as shown in Fig 20 (S1 Data) can effectively quantify the endothermic cooling effect.

The maximum temperature change rate -0.23°C/s of measuring point 2 and its average temperature drop rate -0.08°C/s were higher than other measuring points, and two values of measuring points 1 and 3 were relatively high, indicating that the temperature of measuring points close to the nozzle after the endothermic cooling started to approach the other measuring points with the higher drop to achieve the fusion state. The moment of maximum temperature change rate at each measuring point ranged from 140 s to 160 s, and then the temperature change rate decreased to a stable state subsequently. In addition, the temperature change rate of each measuring point had an alternating pattern of increase and decrease over time, because the liquid was continuously jetted in batches, its gasification effect presented the characteristics of continuous wave after wave, just like the experimental result of gasification jet. Therefore, the fluctuation in the capacity of endothermic cooling for liquid per share also corresponded to the transformation process of forced movement and expansion as well as diffusion. The above mentioned characteristic quantities of temperature change in 200 s on the liquid gasification jet path are shown in Table 3.

**Table 3:** Characteristic quantities of temperature change in 200 s on liquid gasification jet path.

Measuring point	1	2	3	4	5	6
Highest temperature *T*_*H*_/°C	23.3	24.1	24.4	24.3	24.3	24.5
Lowest temperature *T*_*L*_/°C	17.4	15.8	15.6	15.4	15.2	14.9
Temperature change range *T*_*Δ*_/°C	5.9	8.3	8.8	8.9	9.1	9.6
Maximum temperature change rate *r*_*M*_/°C/s	-0.17	-0.23	-0.21	-0.12	-0.13	-0.20
Average temperature drop rate *r*_*A*_/°C/s	-0.06	-0.08	-0.07	-0.05	-0.05	-0.05
Moment of temperature dropping firstly *t*_*D*_/s	110	110	90	30	20	30
Moment of maximum temperature change rate *t*_*M*_/s	140	140	140	150	150	160

### Flame retardant

When the carbon dioxide in the form of mixture from gas and liquid as well as a small amount of frosty dry ice were jetted toward the drum, immediately all gas including the gasified liquid and solid would cover the fire source. The whole liquid discharge process did not exist ice blocking, the burning was stopped due to isolation and dilution of oxygen. However, in an open environment, the discharge time was long, and the inert medium must be aligned with the fire source for continuous action to extinguish it. If the discharge was suspended or the injection direction deviated from the fire source, it was easy to cause the fire source to reignite. When the pressure was less than 1.80 MPa, the spool of pressure regulating device would start the action to pressurize for tank continuously, and amount of gas could be adjusted reasonably to maintain the pressure at this set point until the later stage of discharge.

### Continuous discharge

**Fig 21:**
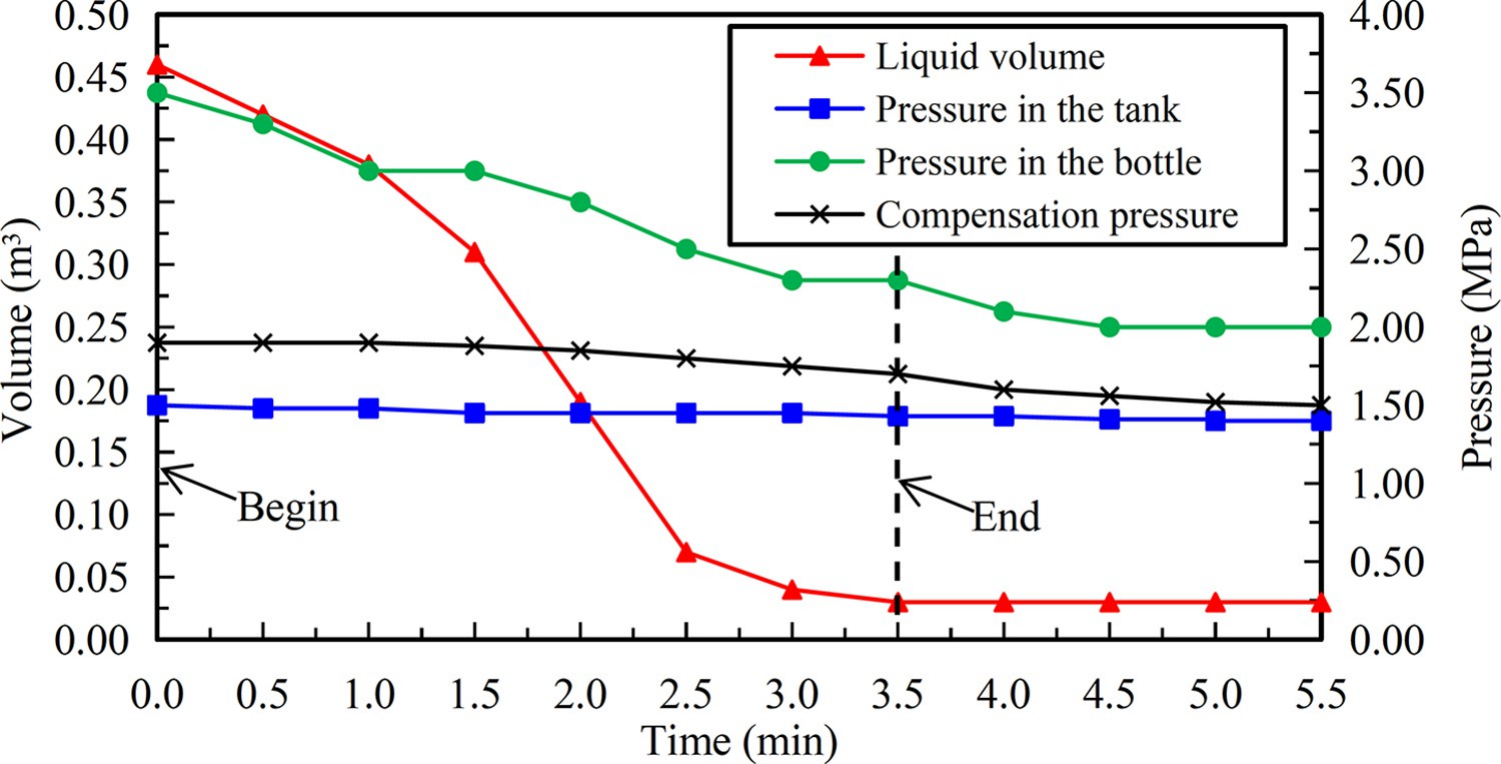
Trends of late liquid volume and pressure indicators.

**Fig 22:**
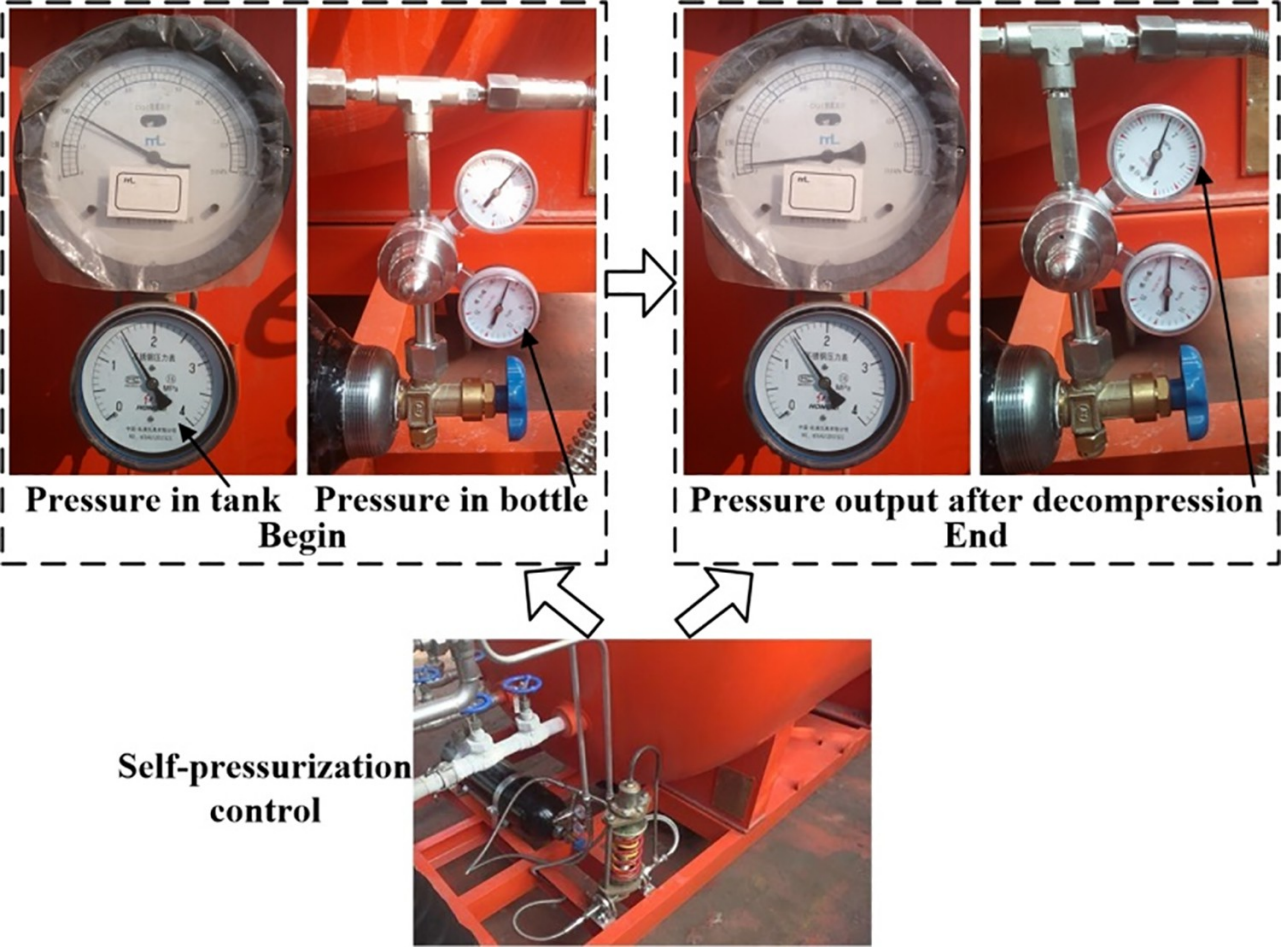
Test of continuous discharge.

The subsequent trends of various parameters are shown in Fig 21 (S1 Data). At the initial moment, the liquid volume and pressure in the tank were 0.46 m^3^ and 1.50 MPa respectively, the pressure in the nitrogen cylinder was 3.50 MPa. In the course of declining liquid volume in the tank, each pressure indicator had been showing a gradual decline with the change of time. At 3.5 min, no liquid carbon dioxide was discharged at the end of the pipe, and the liquid volume was only 0.03 m^3^. In addition, the pressure in the tank was reduced to 1.43 MPa, and the pressure in the nitrogen bottle was depleted to 2.30 MPa from 3.50 MPa. After that, the liquid volume in the tank remained unchanged, and the pressure in the nitrogen cylinder was stabilized at 2.00 MPa quickly. Therefore, based on a comprehensive analysis of actual discharge conditions and changing trends of various parameters, it was confirmed that the discharge was completed at 3.5 min, and the net discharge rate was about 99%. If the pressure in the tank did not exceed 1.50 MPa at all times, the liquid flow rate was 0.12 m^3^/min calculated from the difference between the initial and the end of liquid volume, then the liquid of 1.97 m^3^ would take 16.4 min to be completely discharged after considering the net discharge rate. However, about 3/4 of liquid in the tank was discharged at the pressure of no less than 1.50 MPa, and due to the influence of self-pressurization regulation, it was basically no less than 1.80 MPa, and the time required for the pressure to decrease from 1.80 MPa to 1.50 MPa was relatively short. In addition, the flow velocity of liquid transported by pipeline is determined by the delivery pressure, and the two are in a positive proportion relationship, while the flow rate is equal to the product of flow velocity and pipeline cross-sectional area, so the relationship between pressure and flow rate under the same pipe network diameter is also in a positive proportion. That is, the higher the working pressure, the greater the discharge flow rate, then the shorter the discharge time for a quantitative liquid volume. Therefore, after the liquid is filled, the actual time to completely discharge should not exceed 12.0 min, which is consistent with the total time of 10.5 min for various tests that tank 2 had participated in. The ground liquid gasification injection method uses an air temperature gasifier with the flow rate of 500 m^3^/h, which is a relatively large specification. After calculation, the flow rate of natural gasification after the liquid direct injection is 12 times more than the gas perfusion after liquid gasification on the ground, and the latter has efficiency losses. The field condition of continuous discharge and the values of each instrument in the process are shown in Fig 22.

## Conclusion

Through research on the performance influencing factors for new mobile approach after the design and development of versatile and miniaturized equipment, our major findings can be summarized as following points:

1. The storage tank of vacuum degree 1.5 Pa and air leakage rate 7.5×10^−7^ Pa·m^3^/s with the form of 200 mm vacuum interlayer supplemented by powder can ensure that the daily evaporation rate is only 1/3 of national standard 0.30% at about 20°C, and this value is increased by 3 times when the temperature rises to about 30°C, so the effective volume of liquid inside the 2 m^3^ tank is not less than 1.69 m^3^ after 48 days, then its daily evaporation rate will only increase to about 0.55% after 3 years. This interlayer thickness reaching 2 m^3^ standard is design limit of miniaturized structure used for small and medium sized mine’s roadway section and transport conditions. As the guaranteed equipment of safe production should always be in combat readiness, its standardized research of structural parameters is recommended to carry out in order to save daily maintenance costs of filling;
2. The pneumatic pressurization of dual mode and steel structure are suitable for disaster environment under coal mine. 25 L nitrogen can match emissions of 2 m^3^ liquid carbon dioxide, the liquid discharge time for a single tank is guaranteed about 10.5 min, then the calculation and verification of nitrogen consumption are recommended for research on serialized equipment, and the setting for optimal threshold of filling pressure should be tested and analyzed;
3. The liquid carbon dioxide discharged from the tank is affected weakly by the ambient temperature at the maximum length of 100 m in the pipe, and its physical state is constant. Further, it is necessary to study the insulation technical measures of pipe surface after prolonging the delivery distance;
4. In the initial stage after the liquid in the pipe enters the external environment under 2.20 MPa, the temperature farther from the nozzle can first begin to drop at 20 s, which is 90 s earlier than the temperature drop near the nozzle, and the maximum temperature change range will reach 9.6°C at 200 s. In addition, the maximum temperature change rate at different positions on the liquid gasification jet path during 140 s to 160 s is from -0.12°C/s to -0.23°C/s, and the average temperature drop rate after the temperature starts to drop reaches from -0.05°C/s to -0.08°C/s;
5. The method of 5th order polynomial fitting and keeping 12 decimal places is used to generate the temperature calculation formulas of different positions on liquid gasification jet path with time, the maximum decisive coefficient reaches 0.9930, and the maximum temperature deviation between the fitted curve value and the measured value at every moment does not exceed 0.9204°C, then the crude calculation formula of temperature changing with time within the liquid jet range is formed to reflect the continuous cooling characteristics of the liquid at different initial temperatures;
6. Although the effect of covering and extinguishing fire source in an open environment is poor for inert medium, the mobile approach will be inevitable to extinguish fires because of decrease in oxygen content. If this zone is transformed into a fully enclosed space, the inerting effect will be more ideal.

## Supporting information

S1 Data Results and discussion. (ZIP)
