# Supplementary material for: Investigation on a mobile fire extinguishing approach using liquid carbon dioxide as inert medium for underground mine
Source: PLoS One. 2024 Apr 15;19(4):e0299940. doi: 10.1371/journal.pone.0299940 (PMC11018275; doi:10.1371/journal.pone.0299940)
Supplement: S1 Data — (ZIP) [file pone.0299940.s001.zip › S1 Data. Results and discussion/S1 Data. Results and discussion.docx]

Investigation on a mobile fire extinguishing approach using liquid carbon dioxide as inert medium for underground mine

S1 Data. Results and discussion

**Insulation storage**

The test data trends of insulation storage performance are shown in Fig 15, and the original test data used for drawing is shown in Table 4.

(a) Group 1 test

(b) Group 2 test

**Fig 15. Change of liquid volume and pressure in the tank.**

**Table 4.** **Test data for change of liquid volume and pressure in the tank.**

| **Group number** | **Time (day)** | **Liquid volume (m^3^)** | **Pressure in the tank (MPa)** |
| --- | --- | --- | --- |
| 1 | 0 | 1.9750 | 1.90 |
|  | 3 | 1.9750 | 1.90 |
|  | 6 | 1.9600 | 2.00 |
|  | 9 | 1.9750 | 2.10 |
|  | 12 | 2.0275 | 2.18 |
|  | 15 | 1.9375 | 2.20 |
|  | 18 | 1.9300 | 2.19 |
|  | 21 | 1.9450 | 2.16 |
|  | 24 | 1.9300 | 2.18 |
|  | 27 | 1.9300 | 2.19 |
|  | 30 | 1.9205 | 2.19 |
|  | 33 | 1.9205 | 2.19 |
| 2 | 0 | 1.7590 | 2.00 |
|  | 6 | 1.7400 | 2.20 |
|  | 12 | 1.7136 | 2.20 |
|  | 18 | 1.6520 | 2.20 |
|  | 24 | 1.6300 | 2.20 |
|  | 30 | 1.6080 | 2.20 |
|  | 36 | 1.5085 | 2.20 |
|  | 42 | 1.4970 | 2.20 |
|  | 48 | 1.4970 | 2.20 |
|  | 54 | 1.4395 | 2.20 |
|  | 60 | 1.3705 | 2.20 |
|  | 66 | 1.3705 | 2.20 |

The data of insulation storage performance under 15 ℃ to 32 ℃ for 35 days after 3 years are shown in Fig 16, and the original test data used for drawing is shown in Table 5.

**Fig 16. Change of liquid volume and pressure in the tank put into use for 3 years.**

**Table 5.** **Test data for change of liquid volume and pressure in the tank put into use for 3 years.**

| **Time (day)** | **Liquid volume (m^3^)** | **Pressure in the tank (MPa)** |
| --- | --- | --- |
| 0 | 0.5800 | 2.00 |
| 1 | 0.5500 | 2.10 |
| 2 | 0.5500 | 2.13 |
| 3 | 0.5200 | 2.12 |
| 4 | 0.5200 | 2.12 |
| 5 | 0.5200 | 2.15 |
| 6 | 0.5200 | 2.15 |
| 7 | 0.5000 | 2.15 |
| 8 | 0.5000 | 2.18 |
| 9 | 0.4800 | 2.18 |
| 10 | 0.4800 | 2.18 |
| 11 | 0.4800 | 2.20 |
| 12 | 0.4600 | 2.20 |
| 13 | 0.4200 | 2.18 |
| 14 | 0.3910 | 2.18 |
| 15 | 0.4200 | 2.15 |
| 16 | 0.4600 | 2.15 |
| 17 | 0.4100 | 2.18 |
| 18 | 0.4000 | 2.18 |
| 19 | 0.3460 | 2.20 |
| 20 | 0.3370 | 2.20 |
| 21 | 0.3370 | 2.20 |
| 22 | 0.3280 | 2.20 |
| 23 | 0.3190 | 2.20 |
| 24 | 0.3010 | 2.18 |
| 25 | 0.2920 | 2.18 |
| 26 | 0.2650 | 2.20 |
| 27 | 0.2650 | 2.20 |
| 28 | 0.2380 | 2.20 |
| 29 | 0.2380 | 2.20 |
| 30 | 0.2200 | 2.20 |
| 31 | 0.2200 | 2.20 |
| 32 | 0.2130 | 2.20 |
| 33 | 0.2130 | 2.20 |
| 34 | 0.1920 | 2.20 |
| 35 | 0.1920 | 2.20 |

**Endothermic cooling**

The difference from endothermic cooling effect at each measuring point after liquid gasification jet under 2.20 MPa is shown in Fig 19, and the original test data used for drawing is shown in Table 6.

**Fig 19. Change curves of temperature in 200 s on liquid gasification jet path.**

**Table 6.** **Test data for change curves of temperature in 200 s on liquid gasification jet path.**

| **Time (s)** | **Temperature (℃)** | | | | | |
| --- | --- | --- | --- | --- | --- | --- |
|  | **Point 1** | **Point 2** | **Point 3** | **Point 4** | **Point 5** | **Point 6** |
| 0 | 23.0 | 23.7 | 24.3 | 24.3 | 24.3 | 24.5 |
| 10 | 23.0 | 23.7 | 24.3 | 24.3 | 24.3 | 24.5 |
| 20 | 23.1 | 23.8 | 24.4 | 24.3 | 24.2 | 24.5 |
| 30 | 23.1 | 23.8 | 24.3 | 24.2 | 24.2 | 24.4 |
| 40 | 23.1 | 23.8 | 24.3 | 24.2 | 24.2 | 24.4 |
| 50 | 23.2 | 23.8 | 24.3 | 24.2 | 24.1 | 24.3 |
| 60 | 23.2 | 23.8 | 24.3 | 24.2 | 24.1 | 24.3 |
| 70 | 23.2 | 23.8 | 24.3 | 24.1 | 24.1 | 24.2 |
| 80 | 23.2 | 24 | 24.3 | 24.1 | 24.1 | 24.2 |
| 90 | 23.3 | 24.1 | 24.2 | 24.1 | 24.1 | 24.2 |
| 100 | 23.3 | 24.1 | 24.0 | 24.0 | 24.0 | 24.1 |
| 110 | 23.2 | 23.7 | 23.4 | 23.6 | 23.6 | 23.6 |
| 120 | 22.9 | 22.8 | 22.2 | 22.6 | 22.5 | 22.1 |
| 130 | 22.9 | 22.8 | 22.2 | 21.5 | 21.2 | 20.7 |
| 140 | 21.2 | 20.5 | 20.1 | 20.4 | 20.4 | 19.7 |
| 150 | 21.2 | 20.5 | 20.1 | 19.2 | 19.1 | 19.7 |
| 160 | 20.5 | 19.4 | 19.0 | 18.0 | 18.1 | 17.7 |
| 170 | 19.5 | 18.3 | 18.2 | 17.1 | 17.1 | 16.5 |
| 180 | 18.9 | 17.5 | 17.2 | 17.1 | 17.1 | 16.5 |
| 190 | 18.1 | 16.6 | 16.3 | 16.2 | 16.1 | 15.6 |
| 200 | 17.4 | 15.8 | 15.6 | 15.4 | 15.2 | 14.9 |

The temperature change rate in 200 s on the liquid gasification jet path as shown in Fig 20, and the original calculation data used for drawing is shown in Table 7.

**Fig 20.** **Temperature change rate curves in 200 s on liquid gasification jet path.**

**Table 7.** **Calculation data for temperature change rate curves in 200 s on liquid gasification jet path.**

| **Time (s)** | **Temperature change rate (℃/s)** | | | | | |
| --- | --- | --- | --- | --- | --- | --- |
|  | **Point 1** | **Point 2** | **Point 3** | **Point 4** | **Point 5** | **Point 6** |
| 0 | 0 | 0 | 0 | 0 | 0 | 0 |
| 10 | 0 | 0 | 0 | 0 | 0 | 0 |
| 20 | 0.01 | 0.01 | 0.01 | 0 | -0.01 | 0 |
| 30 | 0 | 0 | -0.01 | -0.01 | 0 | -0.01 |
| 40 | 0 | 0 | 0 | 0 | 0 | 0 |
| 50 | 0.01 | 0 | 0 | 0 | -0.01 | -0.01 |
| 60 | 0 | 0 | 0 | 0 | 0 | 0 |
| 70 | 0 | 0 | 0 | -0.01 | 0 | -0.01 |
| 80 | 0 | 0.02 | 0 | 0 | 0 | 0 |
| 90 | 0.01 | 0.01 | -0.01 | 0 | 0 | 0 |
| 100 | 0 | 0 | -0.02 | -0.01 | -0.01 | -0.01 |
| 110 | -0.01 | -0.04 | -0.06 | -0.04 | -0.04 | -0.05 |
| 120 | -0.03 | -0.09 | -0.12 | -0.10 | -0.11 | -0.15 |
| 130 | 0 | 0 | 0 | -0.11 | -0.13 | -0.14 |
| 140 | -0.17 | -0.23 | -0.21 | -0.11 | -0.08 | -0.10 |
| 150 | 0 | 0 | 0 | -0.12 | -0.13 | 0 |
| 160 | -0.07 | -0.11 | -0.11 | -0.12 | -0.10 | -0.20 |
| 170 | -0.10 | -0.11 | -0.08 | -0.09 | -0.10 | -0.12 |
| 180 | -0.06 | -0.08 | -0.10 | 0 | 0 | 0 |
| 190 | -0.08 | -0.09 | -0.09 | -0.09 | -0.10 | -0.09 |
| 200 | -0.07 | -0.08 | -0.07 | -0.08 | -0.09 | -0.07 |

**Continuous discharge**

The subsequent trends of various parameters are shown in Fig 21, and the original test data used for drawing is shown in Table 8.

**Fig 21. Trends of late liquid volume and pressure indicators.**

**Table 8.** **Test data for trends of late liquid volume and pressure indicators.**

| **Time**  **(min)** | **Liquid volume**  **(m^3^)** | **Pressure in the tank**  **(MPa)** | **Pressure in the bottle**  **(MPa)** | **Compensation pressure**  **(MPa)** |
| --- | --- | --- | --- | --- |
| 0 | 0.46 | 1.50 | 3.5 | 1.90 |
| 0.5 | 0.42 | 1.48 | 3.3 | 1.90 |
| 1.0 | 0.38 | 1.48 | 3.0 | 1.90 |
| 1.5 | 0.31 | 1.45 | 3.0 | 1.88 |
| 2.0 | 0.19 | 1.45 | 2.8 | 1.85 |
| 2.5 | 0.07 | 1.45 | 2.5 | 1.80 |
| 3.0 | 0.04 | 1.45 | 2.3 | 1.75 |
| 3.5 | 0.03 | 1.43 | 2.3 | 1.70 |
| 4.0 | 0.03 | 1.43 | 2.1 | 1.60 |
| 4.5 | 0.03 | 1.41 | 2.0 | 1.56 |
| 5.0 | 0.03 | 1.40 | 2.0 | 1.52 |
| 5.5 | 0.03 | 1.40 | 2.0 | 1.50 |
